# MYB57 transcriptionally regulates MAPK11 to interact with PAL2;3 and modulate rice allelopathy

**DOI:** 10.1093/jxb/erz540

**Published:** 2019-12-07

**Authors:** Changxun Fang, Luke Yang, Weisi Chen, Lanlan Li, Pengli Zhang, Yingzhe Li, Haibin He, Wenxiong Lin

**Affiliations:** 1 Fujian Provincial Key Laboratory of Agroecological Processing and Safety Monitoring, College of Life Sciences, Fujian Agriculture and Forestry University, Fuzhou, China; 2 Key Laboratory of Ministry of Education for Genetics, Breeding and Multiple Utilization of Crops, Fujian Agriculture and Forestry University, Fuzhou, China; 3 Key Laboratory of Crop Ecology and Molecular Physiology (Fujian Agriculture and Forestry University), Fujian Province University, Fuzhou, China; 4 Hong Kong Baptist University

## Abstract

Rice allelopathy is a natural method of weed control that is regarded as an eco-friendly practice in agroecology. The allelopathic potential of rice is regulated by various genes, including those that encode transcription factors. Our study characterized a MYB transcription factor, OsMYB57, to explore its role in the regulation of rice allelopathy. Increasing the expression of *OsMYB57* in rice using the transcription activator VP64 resulted in increased inhibitory ratios against barnyardgrass. The gene expression levels of *OsPAL*, *OsC4H*, *OsOMT*, and *OsCAD* from the phenylpropanoid pathway were also up-regulated, and the content of l-phenylalanine increased. Chromatin immunoprecipitation incorporated with HiSeq demonstrated that OsMYB57 transcriptionally regulated a mitogen-activated protein kinase (OsMAPK11); in addition, OsMAPK11 interacted with OsPAL2;3. The expression of *OsPAL2;3*was higher in the allelopathic rice PI312777 than in the non-allelopathic rice Lemont, and *OsPAL2;3* was negatively regulated by Whirly transcription factors. Moreover, microbes with weed-suppression potential, including *Penicillium* spp. and *Bacillus* spp., were assembled in the rhizosphere of the rice accession Kitaake with increased expression of OsMYB57, and were responsible for phenolic acid induction. Our findings suggest that OsMYB57 positively regulates rice allelopathy, providing an option for the improvement of rice allelopathic traits through genetic modification.

## Teaser

MYB57 positively regulates MAPK11, and MAPK11 interacts with PAL2;3 to modulate rice allelopathy. The allelopathic rice PI312777 has a higher transcriptional ability of *PAL2;3* than the non-allelopathic rice Lemont.

## Introduction

Allelopathy is a biological phenomenon, typical of certain plants, algae, and microorganisms (Reigosa *et al.*, 2006), that involves inhibitory or stimulatory effects of one organism on another. This phenomenon can influence organism growth, survival, and reproduction through the secretion of allelochemicals or their release into the environment (Rice, 1984). Allelochemicals belong to the secondary plant metabolites, and the role of several groups of secondary metabolites, such as phenolic acids and terpenoids, in allelopathy has been documented (Maqbool *et al.*, 2013; Soltys *et al.*, 2013; Einhellig, 2018). They are predominantly derived from the phenylpropanoid metabolic and isoprenoid biosynthetic pathways and their associated branch paths (Verpoorte, 2000).

In rice, allelopathic effects were first recorded by Dilday *et al.* (1989) during a field experiment that presented different rice accessions with ~50–90% inhibitory ratios against ducksalad [*Heteranthera limosa* (Sw.) Willd.]. Since that study, several studies have screened potential allelopathic rice accessions and have separated and identified rice allelochemicals (Dilday *et al.*, 1994; Olofsdotter *et al.*, 1995; Chung *et al*., 2001, 2006). To date, various studies have shown that phenolic acids such as ferulic acid (FA), cinnamic acid (CA), *p*-hydroxybenzoic acid (HA), and momilactone B, which is a diterpenoid, have allelopathic activity against barnyardgrass (Kato-Noguchi, 2004, 2011; Seal *et al.*, 2004; Bi *et al.*, 2007; Kato-Noguchi and Ino, 2005; Khanh *et al.*, 2007; He *et al.*, 2012*a,b*). The knockout or silencing of relevant genes participating in the diterpene or phenolic acid pathways, such as copalyl diphosphate synthase 4 (*OsCPS4*), kaurene synthase-like 4 (*OsKSL4*), and phenylalanine ammonia lyase (*OsPAL*), results in weakened allelopathic activity of donor rice on barnyardgrass. This weakened activity is attributed to reduced contents of momilactones or phenolic acid in root exudates (Xu *et al.*, 2012; Fang *et al.*, 2013). Studies have documented that rhizospheric microorganisms have indispensable roles in the process of allelopathy (Inderjit, 2005; Fang *et al.*, 2015; Li *et al.*, 2015; Niro *et al.*, 2016; Wu *et al.*, 2019). Specifically, *Sphingobium* spp. from the rhizosphere of peanut (*Arachis hypogaea*) functioned in the biodegradation of pterostilbene produced by the plants, suggesting that bacterial interference may play a role in plant allelopathy (Yu *et al.*, 2019). Exogenous vanillin influenced the bacterial and fungal communities of the rhizosphere of cucumber (*Cucumis sativus* L.) seedlings (Jia *et al.*, 2018; Zhang *et al.*, 2018). Vanillic acid also changed the total fungal community in the cucumber seedling rhizosphere, and the *Trichoderma* and *Fusarium* spp. communities showed different responses to vanillic acid (Chen *et al.*, 2018). In rice, phenolic acids secreted from the root tissue were found to interact with specific microorganisms in the rice rhizosphere (e.g. *Myxococcus xanthus*) to increase the inhibition of weeds. This inhibition indicates the vital role of the interaction of *M. xanthus* and phenolic acids in rice allelopathy in the field (Fang *et al.*, 2015). These compounds are known to be derived from the phenylpropanoid metabolic pathway.

Previous studies have shown that MYB transcription factors function in the regulation of genes involved in the plant phenylpropanoid metabolic pathway (Yang *et al.*, 2012; Liu *et al.*, 2015*a*). MYB transcription factors in plants can be classified into four types depending on the number of adjacent repeats in the MYB domain, namely 1R-MYB, R2R3-MYB, 3R-MYB, and 4R-MYB, which contain one, two, three, and four MYB repeats, respectively (Dubos *et al.*, 2010). Among these four types, R2R3-MYB factors are the main components of the MYB family and play a role in the regulation of genes involved in secondary metabolism (Liu *et al.*, 2015*a*). For example, constitutively expressed *Zm*MYB-IF35 promotes the accumulation of ferulic and chlorogenic acids in maize (Dias and Grotewold, 2003). Similarly, the genes *StAN1*, *StMTF1*, and *PAP1* in potato and *AtMYB12* in Arabidopsis, which were constitutively expressed in tobacco, potato, tomato, and snow lotus (*Saussurea involucrata*), respectively, resulted in an increase in the chlorogenic acid content (Luo *et al.*, 2008; Rommens *et al.*, 2008; Payyavula *et al.*, 2013; Qiu *et al.*, 2013). In grape, the overexpression of *VvMYB5a*, which encodes a protein belonging to a small subfamily of R2R3-MYB transcription factors, induced strong accumulation of various phenolic compounds. This finding suggests that *VvMYB5a* is involved in the control of different branches of the phenylpropanoid pathway in grapevine (Deluc *et al.*, 2006). In *Arabidopsis thaliana*, subgroup 7 of the R2R3-MYB gene family, which includes MYB11, MYB12, and MYB111, plays a role in the regulation of the gene expression of *chalcone synthase*, *chalcone isomerase*, *flavanone 3-hydroxylase*, and *flavonol synthase1*, all of which are involved in flavonoid biosynthesis, thereby controlling flavonol accumulation in the plant (Stracke *et al.*, 2007).

In rice, genome-wide analysis identified at least 185 *MYB* genes, including genes encoding 70 1R-MYBs, 109 R2R3-MYBs, 5 3R-MYBs, and 1 4R-MYB (Chen *et al.*, 2006; Katiyar *et al.*, 2012). The overexpression of the R2R3-type *OsMYB60* resulted in twisted leaf blades in transgenic rice (Liu *et al.*, 2018). Another R2R3-type transcription factor, OsMYB1, was identified in response to phosphate starvation (Gu *et al.*, 2017). Yang *et al.* (2012) identified a R2R3-type MYB gene, *OsMYB2*, that is involved in the tolerance of salt, cold, and dehydration in rice. To the best of our knowledge, however, aside from these studies, there has been no investigation of the involvement of R2R3-type MYBs in the regulation of rice allelopathy, despite the potential role of R2R3 MYBs in the regulation of secondary pathways. Our previous studies screened a set of VP64 transcription factor transgenic rice lines of the accession Kitaake, constructed by Zhao *et al.* (2015), to investigate their weed-suppression capacity. An increased weed-suppression ability was found in a VP64-activated R2R3 MYB transcription factor *OsMYB57* transgenic line. Wang *et al.* (2016) used these transgenic lines to investigate the transcription factor that possibly increased rice grain yield, and a 35% increase in grain yield was found in the *OsMYB1R1*-VP64 transformed line. The *UL48* gene-encoded viral protein 16 (VP16) is a transcription activation domain from the human herpes simplex virus, which is one of the best-known transcription regulatory domains (Sadowski *et al.*, 1988). The C terminal of the VP16 protein maintains an 11-amino acid peptide with transcription activation function, and four repeats of this peptide (VP64) have even stronger activation properties (Yaghmai *et al.*, 2002). Both VP16 and VP64 represent a new strategy for genetic engineering. The activity of transcription factors is significantly increased after they have been fused with VP64, enabling them to play a stronger role in regulating crop growth and development. According to the results of the current study, increasing OsMYB57 expression in rice resulted in larger inhibitory ratios against barnyardgrass, but the regulatory network of OsMYB57 remains unknown. In Arabidopsis, the expression of *MYB57* was induced by jasmonate and was able to promote stamen filament growth (Cheng *et al.*, 2009). However, the coding sequences of OsMYB57 and AtMYB57 share little homology (35.66%), suggesting that OsMYB57 in rice may have a distinct role.

The aim of this study was to clarify the exact role of OsMYB57 in the regulation of rice allelopathy. To reveal the genes that were transcriptionally regulated by OsMYB57, chromatin immunoprecipitation sequencing (ChIP-seq) was conducted to identify the motif of the relevant genes regulated by OsMYB57. Correlations with the rice phenylpropanoid pathway were explored, and the role of OsMYB57 in regulating allelopathy was revealed.

## Materials and methods

### Plant materials

This study used the rice (*Oryza sativa* L. subsp. *japonica*) accession Kitaake and its transgenic lines with increased OsMYB57 expression via a transcriptional activator containing four copies of VP16 (i.e. VP64) (Sadowski *et al.*, 1988; Yaghmai *et al.*, 2002). The allelopathic rice (*O. sativa* L. subsp. *japonica*) accession PI312777 and the non-allelopathic rice (*O. sativa* L. subsp. *japonica*) accession Lemont were also used (Bollich *et al.*, 1985; Fang *et al.*, 2015).

### Rice growth conditions

The seeds of each rice line were treated with 25% NaClO for 30 min for surface sterilization and then soaked in sterilized double-distilled (dd)H_2_O overnight. The imbibed seeds were transferred to an artificial climate incubator at 30 °C to germinate, and germinated seeds were sown on to separate seedling plates and grown until they were at the three-leaf stage. Uniform seedlings with similar growth rates from each line were selected and transplanted to a Styrofoam plate containing 20 equally distributed holes by using a sponge to affix the seedlings and insert them into each hole. The Styrofoam plate was floated in a pot (45 cm × 35 cm × 15 cm) filled with 10 litres of rice culture solution (Fang et al., 2011, 2016), according to a modified technique described by Yoshida (1976). The solution was replaced with fresh solution every week, and its pH was maintained at 5.5–6.0 throughout the experiment.

### Evaluation of the rice allelopathic inhibition of barnyardgrass

To evaluate the allelopathic potential of OsMYB57_VP64_ and Kitaake, the seeds of these two rice lines were again surface sterilized, germinated and sown in agar medium and in the soil. The OsMYB57_VP64_ transgenic rice lines were constructed by Zhao *et al.* (2015). For the agar culture condition experiments, 10 germinated rice seeds were sown on plates (9 cm diameter) containing 200 ml 0.6% solid agar. After 1 week, 10 germinated barnyardgrass seeds were also sown on the plates. For the soil culture condition experiments, five germinated rice seeds were sown in a 0.5 litre beaker (8.5 cm diameter) containing 100 ml of soil. The soil was sandy loam with pH 6.5 and 55.8% moisture content, 53.75  mg kg^–1^ alkali-hydrolysable nitrogen, 11.10 mg kg^–1^ Olsen phosphorus, 72.67 mg kg^–1^ available potassium, and 20.82 g kg^−1^ organic matter. After 1 week, five germinated barnyardgrass seeds were also sown in the beaker, and both the plates and beakers were placed in an artificial climate incubator under 12 h/12 h (day/night) photoperiod, 10 000 lux, and 75% relative humidity. A total of 20 mono-cultured barnyardgrass seeds grown on a plate or 10 mono-cultured barnyardgrass seeds in a beaker were used as controls. Both the treatment and control were performed in triplicate. The barnyardgrass seeds were allowed to grow for 1 week, after which the root length, plant height, and fresh weight of barnyardgrass co-cultured with OsMYB57_VP64_ or Kitaake and the control mono-cultured barnyardgrass were measured to determine the allelopathic potential of rice on barnyardgrass (Fang *et al.*, 2013).

### Detection of *Os*MYB57 expression in VP64-derived transformed rice and wild-type rice

The top second leaf of the VP64-derived OsMYB57-transformed Kitaake rice line (OsMYB57_VP64_) and its wild type (WT) were sampled to extract the total proteins for western blotting. Specific antibodies to OsMYB57 and goat anti-rabbit IgG conjugated to horseradish peroxidase were successively used in western blots to detect the levels of OsMYB57 expression in OsMYB57_VP64_ and WT Kitaake.

### Identification of the secondary metabolites from root tissues of OsMYB57_VP64_ and Kitaake

Rice allelochemicals are mainly derived from plant secondary metabolites. To investigate the compounds from the OsMYB57_VP64_ and Kitaake rice lines, the two rice lines were co-cultured with barnyardgrass for 7 days. The root tissues of the two rice lines were then harvested and ground individually, and the secondary metabolites were extracted using methanol. The crude extract was then extracted using chloroform, vacuum dried, and derivatized for identification by GC-QqQ mass spectroscopy [Shimadzu (China) Co., Ltd].

### Determination of population numbers of microorganisms from rhizospheric soil of rice

To investigate the differences in the populations of rhizospheric microorganisms from the OsMYB57_VP64_ and Kitaake rice lines, the DNA from the rhizospheric soil of the two rice lines was extracted using a BioFast Soil DNA Extraction Kit (Hangzhou Bioer Technology Co., Ltd). Quantitative PCR (qPCR) was conducted to determine the population numbers of bacteria, fungi, and myxobacteria in the rhizosphere of the two rice lines, based on Fang *et al.* (2013, 2015).

### Isolation of rhizospheric microbes and evaluation of their allelopathic potential against barnyardgrass

Rhizospheric soil samples from OsMYB57_VP64_ and Kitaake (5 g each) were resuspended in 100 ml of sterilized ddH_2_O and shaken at 1000 rpm at 37 °C for 1.5 h, after which the mixture was incubated at room temperature for 10 min. Next, the supernatant was serially diluted from 10-fold to 10^7^-fold and spread on to beef extract peptone agar (BPA) medium and potato dextrose agar (PDA) medium using the plate smearing method. BPA was used for bacterial cultures and PDA was used for fungal cultures. The BPA and PDA medium plates were placed in an incubator at 37 °C and 28 °C, respectively. Colony growth on the media was recorded every 24 h for 1 week. The colonies were then isolated and purified to obtain single strains. The purified bacteria and fungi were further cultured in beef extract peptone liquid medium and potato dextrose liquid medium, and the fermentation broths were collected and used to evaluate their allelopathic potential against barnyardgrass. Barnyardgrass co-cultured in the medium was used as a control.

### Identification of allelopathic bacterial and fungal species

Bacteria and fungi with allelopathic potential were cultured for genomic DNA extraction. An EasyPure Bacteria Genomic DNA Kit (Transgen Biotech) was used for bacterial genomic DNA extraction, and the cetyltrimethylammonium bromide method was used for fungal genomic DNA extraction. Genomic DNA was used for PCR amplification; the primers 27F (5′-AGAGTTTGATCCTGGCTCAG-3′) and 1492 R (5′-GGTTACCTTGTTACGACTT-3′) were used for amplification of bacterial DNA, and ITS1 (5′-TCCGTAGGTGAACCTGCGC-3′) and ITS4 (5′-CAGGAAACAGCTATGACC-3′) were used to amplify fungal DNA. The PCR products were purified and then sequenced at BioSune Biotechnology Co., Ltd (Shanghai). The sequence was aligned in GenBank using BLASTn to identify similarities and homologous strains.

### Induction of microbial growth with phenolic acids

Three phenolic acids, CA (0.0066 μmol l^–1^), FA (0.0031 μmol l^–1^), and HA (0.168 μmol l^–1^), were added to the culture medium to detect the induction effect of phenolic acids on the growth of *Bacillus* and *Pseudomonas* spp. The culture was sampled at 6, 12, 18, 24, and 30 h for *Bacillus* spp. and 12, 24, 36, 48, 60, 72, and 84 h for *Pseudomonas* spp. to determine the OD_600_ value.

### Determination of genes regulated by *Os*MYB57 using ChIP-seq and ChIP-qPCR

To investigate the genes potentially regulated by OsMYB57, chromatin immunoprecipitation (ChIP) was used to obtain the chromatin DNA fragment that bound to OsMYB57. The chromatin DNA was isolated from the leaves of Kitaake rice using 1% formaldehyde to crosslink and then isolate the nuclei and chromatin. The chromatin was sheared into 200–500 bp fragments, subjected to immunoprecipitation using 20 μg of antibody to OsMYB57 or anti-IgG, and collected with DNA beads Protein A (Invitrogen). The IgG antibody bound to these DNA fragments was used as a negative control. The precipitated chromatin was decrosslinked to release the ChIP-DNA, which was purified and quantified (Qubit Fluorometer) for ChIP sequencing (ChIP-seq) library construction and ChIP-qPCR detection. For the ChIP-seq, two libraries (ChIP-DNA and input DNA) were prepared using a library preparation kit (New England Biolabs) according to the manufacturer’s instructions. The libraries were sequenced using an Illumina Genome Analyzer (HiSeq 2000). The library construction and sequencing were performed by Wuhan IGENEBOOK Biotechnology Co., Ltd (http://www.igenebook.com/).

For the ChIP-qPCR, the chromatin DNA fragment bound to OsMYB57 was collected as a template. The sequences of peak 26 and peak 39 from the ChIP-seq and the promoter regions of *OsPAL2;3* (LOC_Os02g41670) and *OsKSL4* (LOC_Os04g10060) were selected to design the primers for ChIP-qPCR (see Supplementary Table S1 at *JXB* online). The DNA fragment bound to the IgG antibody was again used as a negative control for normalization.

### Bimolecular fluorescence complementation and co-immunoprecipitation assays

To investigate the possible interactions among the proteins regulated by OsMYB57, the *OsMAPK11* and *OsPAL2;3* genes were cloned from PI312777. They were then used to construct recombinant vectors with the N-terminal or C-terminal domain of enhanced yellow fluorescent protein (eYFP). These vectors were then transformed into *Agrobacterium* (EHA105) for inoculation into the cytoplasm of tobacco (*Nicotiana benthamiana*) for transient expression assays. Laser scanning confocal microscopy (Leica TCS SP8X DLS, Leica Microsystems Inc.) was used to investigate positive interactions among the target proteins.

Additionally, genes fused with the FLAG tag or eYFP were constructed and transformed into *Agrobacterium* EHA105 for inoculation. The total fraction of soluble proteins of inoculated tobacco leaves was extracted and incubated with FLAG magnetic beads (Sigma) or GFP-Trap agarose (Chromotek) to collect putative interacting proteins. Anti-eYFP and anti-MAPK11 were used in western blotting to detect positive interactions among the proteins.

### Determination of *Os*MYB57, *Os*MAPK11, and *Os*PAL2;3 expression

Seeds of the allelopathic rice PI312777 and the non-allelopathic rice Lemont were sterilized and germinated following the protocol described above for Kitaake, and the seedlings were grown to the three-leaf stage. Seeds of barnyardgrass were treated by using the same method and also grown to the three-leaf stage. Next, 20 uniform PI312777 or Lemont rice seedlings were co-cultured with 20 uniform barnyardgrass seedlings in a 20 litre container. Forty mono-cultured PI312777 or Lemont rice seedlings were prepared as controls under the same conditions as the treatment plants. Both the treatments and the controls were grown in a greenhouse and studied in triplicate. At 1, 3, 5, and 7 days, the top second leaf of the co-cultured or mono-cultured rice was sampled to extract the total fraction of soluble proteins, and western blotting was conducted to detect the protein expression of OsMYB57, OsMAPK11, and OsPAL2;3 using specific antibodies. The protein expression of β-actin was used as a reference.

### Cloning of the *OsPAL2;3* promoter and detection of its activity

The promoter of *OsPAL2;3*, a 3000 bp region upstream of the gene coding DNA sequence (CDS), was amplified from the genomic DNA of PI312777 and Lemont. The promoter was used to replace the △actin2 promoter in pCambia 3301, followed by a 2×FLAG tag and *GFP* gene, to construct recombinant vectors for tobacco leaf and rice protoplast transformations. For tobacco, the transformed leaves were sampled to extract the proteins, and a western blot was performed using anti-FLAG antibody to determine the protein expression level of FLAG fused with GFP. For the rice protoplasts, the intensity of GFP fluorescence was detected using confocal laser scanning microscopy (Nikon C2-ER, Nikon Instruments Co., Ltd).

### DNA pulldown to identify transcription factors binding at the promoter of *OsPAL2;3*

The total fraction of leaf soluble proteins of PI312777 and Lemont was extracted. The promoter region (3000 bp upstream of the CDS) of *OsPAL2;3* was cloned from the DNA of PI312777 and Lemont, respectively, labelled with biotin at the 5′ end, and incubated with the proteins. The interacting proteins were collected using a Dynabeads kilobaseBINDER Kit (Thermo Fisher Scientific) and then separated on 10% SDS-PAGE and identified using Orbitrap Fusion (Thermo Fisher Scientific).

### qPCR detection of the expression levels of transcription factors

The Whirly transcription factors, including *LOC_Os06g05350* (*OsWHY1*) and *LOC_Os02g06370* (*OsWHY2*), and the transcription regulator *histone H4* (*LOC_Os07g36500*), which binds to the promoter region of *OsPAL2;3*, were selected to detect the level of transcripts from PI312777 and Lemont when they were co-cultured with barnyardgrass. The specific primers for *OsWHY1*, *OsWHY2*, and *histone H4* are shown in Supplementary Table S1, and *β-actin* was used as a reference gene for normalization. The relative transcription levels of the genes of the two rice lines cultured together with barnyardgrass for 1, 3, 5, and 7 days were analysed.

### Statistical analysis

All of the experiments were conducted at least three times. The results in the figures are presented as means ±SD. Statistical analyses were based on the Student’s *t*-test.

## Results

### *Os*MYB57 expression level in *Os*MYB57_VP64_ transgenic lines and weed-suppression capacity

**Fig. 1.**
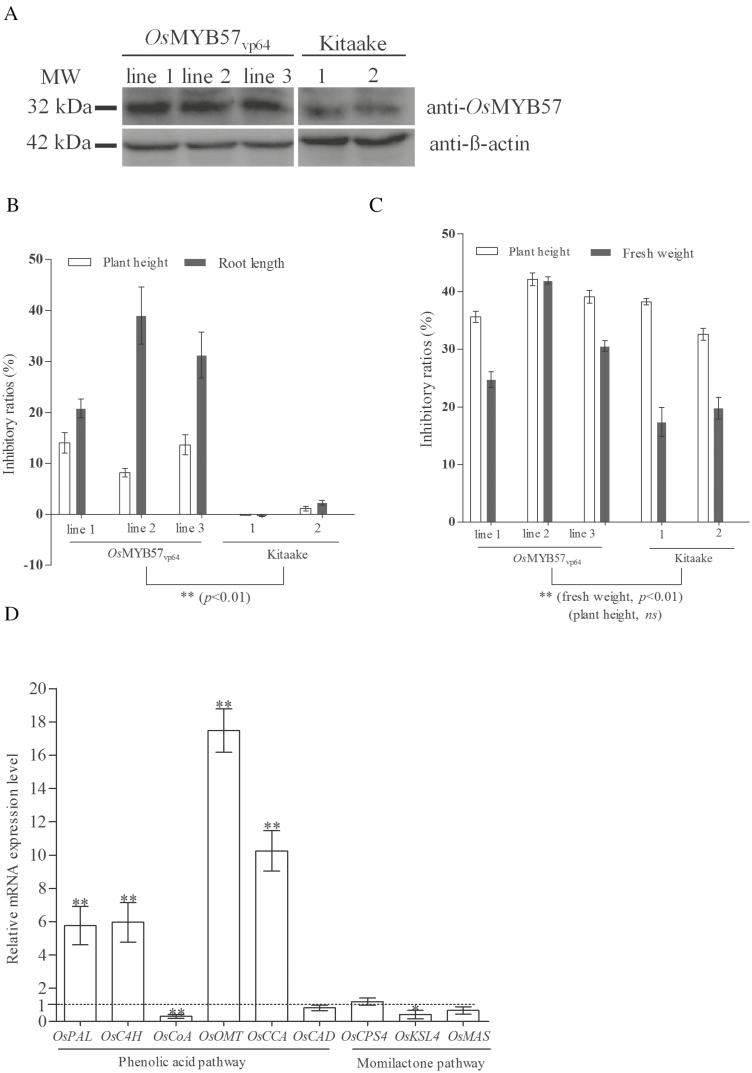
OsMYB57 expression level and the allelopathic inhibition of barnyardgrass by the OsMYB57_VP64_ transgenic rice lines and wild-type (WT) Kitaake rice lines. (A) The protein expression of OsMYB57 was determined from three independent OsMYB57_VP64_ transgenic rice lines and two groups of WT Kitaake, with the protein expression of β-actin as a reference. The allelopathic potential of OsMYB57_VP64_ and Kitaake on barnyardgrass was evaluated in 0.6% agar solid medium (B) and in soil (C). The ratio of rice to barnyardgrass in the co-cultured system was 1:1 (TR), and mono-cultured barnyardgrass was used as a control (CT). Inhibitory ratios (IRs) were calculated as follows: IR=(CT–TR)/CT×100%. IR >0 represents growth inhibition, and IR <0 represents growth promotion. (D) Genes involved in allelochemical synthesis and the associated differences in expression between OsMYB57_VP64_ and Kitaake. To compare the differences in gene expression between the rice lines, the expression of *OsPAL*, *OsC4H*, *OsCoA*, *OsOMT*, *OsCCA*, and *OsCAD*, which are involved in the phenolic acid pathway, and *OsCPS4*, *OsMAS*, and *OsKSL4*, which are involved in the momilactone pathway, was detected in *Os*MYB57_VP64_ and Kitaake (D). The fold change was calculated according to the formula 2^–△△ct^, and the gene expression of *β-actin* was used as a reference for normalization. For each column, significant differences in the gene expression level between OsMYB57_VP64_ and Kitaake are indicated with asterisks: *P<0.05, **P<0.01.

A western blot analysis showed that VP64 led to increased OsMYB57 expression in the transformed rice lines (OsMYB57_VP64_) compared with WT Kitaake (Fig. 1A). The capacity for suppression on the root length of barnyardgrass was enhanced at a ratio of approximately 20–39% for rice with increased OsMYB57 expression levels compared with a ratio of –0.4–2.3% for WT Kitaake in agar culture medium (Fig. 1B). Moreover, when grown in soil, the OsMYB57_VP64_ transgenic lines had a 24.7–41.9% inhibitory ratio against the fresh weight of barnyardgrass, while the WT showed a 17–19% inhibitory ratio (Fig. 1C). The significant enhancement of weed-suppression capacity in the transgenic lines suggests a positive role for OsMYB57 in the regulation of rice allelopathy.

### Changes in the expression of genes participating in the momilactone or phenolic acid pathways

The expression of genes involved in allelochemical synthesis was compared between OsMYB57_VP64_ and Kitaake. Four genes from the phenolic acid pathway, encoding phenylalanine ammonia lyase (*OsPAL*), cinnamate 4-hydroxylase (*OsC4H*), *O*-methyltransferase (*OsOMT*), and cinnamoyl-CoA (*OsCCA*), were up-regulated by a factor of 5.76, 5.96, 17.47, and 10.24, respectively, in OsMYB57_VP64_ compared with Kitaake. The expression of CoA-ligases (*OsCoA*) and cinnamoyl alcohol dehydrogenases (*OsCAD*) was down-regulated in OsMYB57_VP64_ compared with Kitaake. The expression of the genes encoding momilactone-A synthase (*OsMAS*) and *syn*-pimara-7, 15-diene synthase (*OsKSL4*) from the momilactone pathway were down-regulated by a factor of 0.67 and 0.42, respectively, in OsMYB57_VP64_ compared with Kitaake. The expression of copalyl diphosphate synthase (*OsCPS4*) showed no detectable difference between the two rice lines (Fig. 1D). These results suggest that OsMYB57 positively regulated the expression of genes participating in the phenolic acid pathway.

### Changes in the content of secondary metabolites

The determination of the secondary metabolites from the two rice lines identified l-phenylalanine and *p*-toluic acid in the OsMYB57_VP64_ transgenic line, but these metabolites were beyond the lower limit of detection in Kitaake (Supplementary Table S2). l-phenylalanine is the vital precursor substance of phenolic acid and OsPAL catalyses the first step in the production of phenolic acid by using l-phenylalanine as substrate.

### Microbial population numbers from the rhizospheric soil of OsMYB57_VP64_ transgenic lines and WT rice

**Fig. 2.**
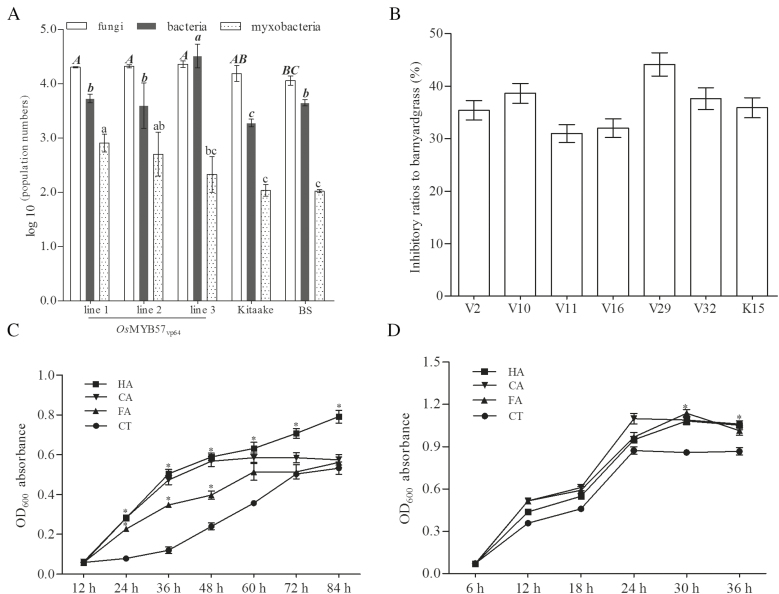
(A) Results of qPCR conducted to determine the population numbers of bacteria, fungi, and myxobacteria from the rhizospheric soil of OsMYB57_VP64_ and Kitaake. For each column, different letters indicate statistical groups that differ significantly. BS, Blank soil sample (soil without rice plants). (B) Allelopathic inhibitory ratios to barnyardgrass from specific microbial strains. Microbial strains from the rhizospheric soil were isolated, and their inhibitory ratios to barnyardgrass were detected using fermentation broth. V2, *Pseudomonas* spp. YXE3-18; V10, *Bacillus* spp. B-15; V11, *Pseudomonas* spp. J3.2C5; V16, *Penicillium aculeatum* strain H23; V29, *Penicillium rubidurum* isolate CY249; V32, *Penicillium rubidurum* isolate CY249; V32, *Streptomyces viridobrunneus* strain SCPE-09; K15, *Streptomyces* spp. FXJ1.430. (C, D) Effects of phenolic acids on the growth of *S. viridobrunneus* strain SCPE-09 (C) and *Bacillus* spp. (D). The growth rates of *S. viridobrunneus* and *Bacillus* spp. were detected after treatment with exogenous cinnamic acid (CA), ferulic acid (FA), and *p*-hydroxybenzoic acid (HA) application and shock culturing. The dynamic OD_600_ value was measured at the indicated times.

Allelopathic potential is well known to be correlated with rhizospheric microbes (Inderjit, 2005); thus, we determined the total amount of bacterial and fungal species in the rhizospheric soil of OsMYB57_VP64_ transgenic lines and WT Kitaake. The rhizospheric soil of OsMYB57_VP64_ transgenic lines had the largest bacterial population compared with the rhizospheric soil of Kitaake and control soil samples without rice plants. The numbers of myxobacteria have been reported to correlate with rice allelopathy (Fang *et al.*, 2015), and these were also higher in the OsMYB57_VP64_ rhizospheric soil than in the rhizospheric soil of Kitaake and soil without rice plants. In addition, the population of fungi from OsMYB57_VP64_ rhizospheric soil was higher than that of the other two samples, but no significant difference was found between OsMYB57_VP64_ and Kitaake (Fig. 2A).

### Isolation of microorganisms from soil and associated weed-suppression capacities

Rhizospheric soil from the OsMYB57_VP64_ transgenic lines and Kitaake was sampled to isolate the culturable micro-organisms. A total of 31 strains were obtained from Kitaake, and 35 strains were isolated from the OsMYB57_VP64_ transgenic lines. The allelopathic potential of these micro-organisms to barnyardgrass was evaluated, and 32 out of 35 strains isolated from the rhizospheric soil of OsMYB57_VP64_ transgenic lines showed weed-suppression capacity (Supplementary Fig. S1A). The inhibition ratios to barnyardgrass were greater than 30% in six strains; the highest inhibition ratio was 44.1% (Fig. 2B). In contrast to the transgenic lines, 16 out of 31 WT strains showed suppression capacity on barnyardgrass (Supplementary Fig. S1B); however, only one strain had an inhibitory ratio of more than 30% (Fig. 2B). These results indicate that OsMYB57_VP64_ in rice stimulated the accumulation of potentially allelopathic microorganisms in the rhizospheric soil. To further investigate these microorganisms, the genomic DNA of each strain with high allelopathic potential was extracted, and the 16S rRNA or ITS region was amplified for sequencing. Two *Pseudomonas* spp., two *Penicillium* spp., one *Bacillus* spp., and one *Streptomyces* spp. were identified from the rhizospheric soil of the OsMYB57_VP64_ transgenic lines. Only one species, a *Streptomyces* spp., was isolated from the WT (Supplementary Table S3; Supplementary Dataset S1). The accumulation of microbes with high allelopathic potential in the rhizospheric soil of OsMYB57_VP64_ rice further indicated the involvement of OsMYB57_VP64_ in the regulation of allelopathic microbial diversity.

### Effect of phenolic acids on microbial growth

The *Streptomyces viridobrunneus* strain SCPE-09 and the *Bacillus* spp. were selected to study the effect of phenolic acids on growth and proliferation. The phenolic acids CA, FA, and HA were added to the culture medium and the effects on the OD_600_ value of the culture were measured. The results showed that, individually, CA, FA, and HA promoted the growth and proliferation of *S. viridobrunneus* strain SCPE-09. After being cultured in the presence of phenolic acid for 24–60 h, the OD_600_ of the phenolic acid-supplemented culture medium was significantly higher than that of the medium without phenolic acids, indicating the growth-inducing effect of phenolic allelochemicals on *S. viridobrunneus* strain SCPE-09 (Fig. 2C). A similar growth-inducing effect was evident for *Bacillus* spp. during the period of 30–36 h (Fig. 2D).

### Genes transcriptionally regulated by *Os*MYB57

**Fig. 3.**
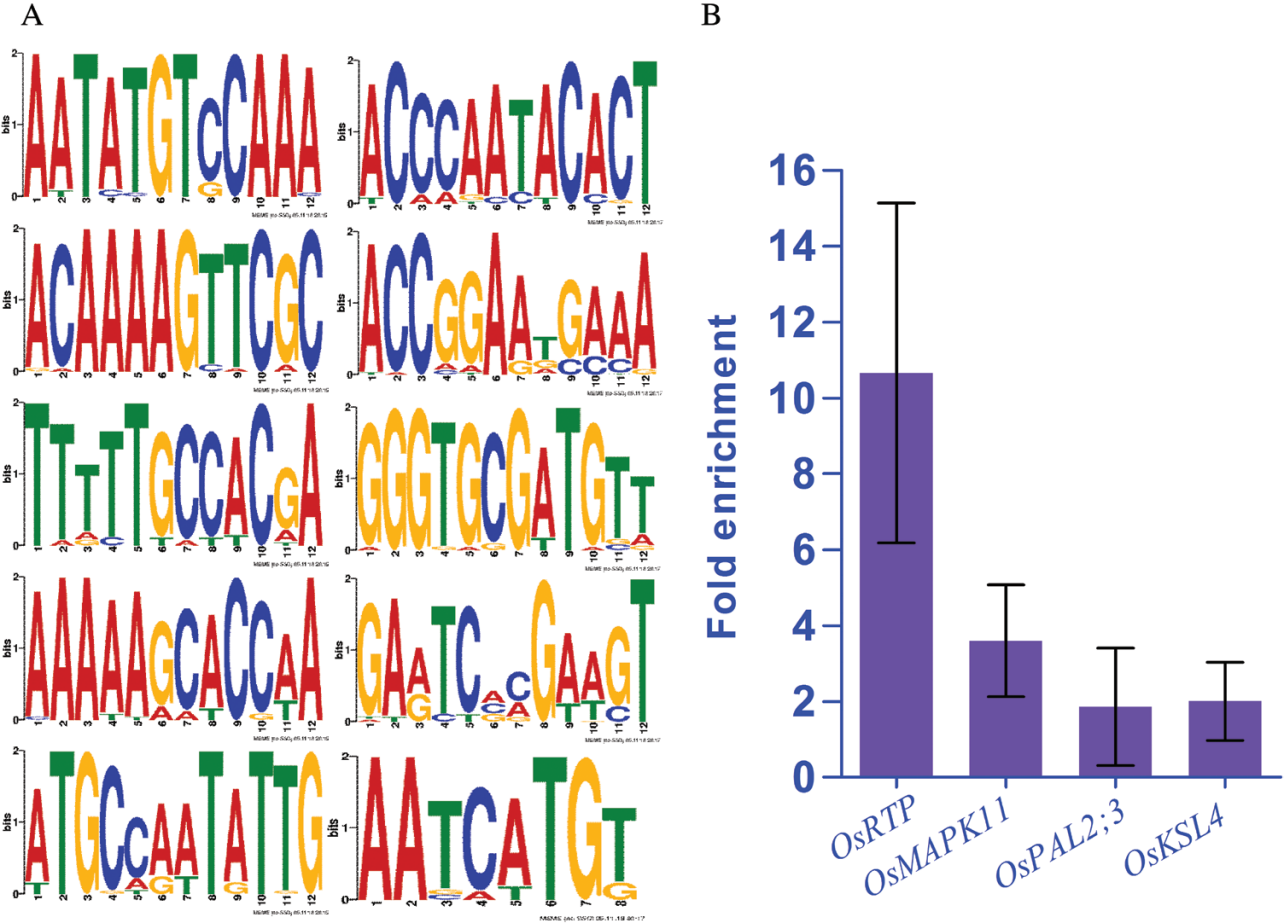
Predicted motifs from the peak sequence of the promoter region for retrotransposon protein and OsMAPK11. (A) MEME (http://meme-suite.org/; Bailey and Elkan, 1994) was used to predict the motif bound by OsMYB57; 10 motifs were predicted in the peaks of the two gene promoter regions of interest, including the genes *retrotransposon protein* and *OsMAPK11*. All 10 motifs were distributed in the sequence of peaks 25 and 39. (B) ChIP-qPCR determination of the fold enrichment on the promoter region of *OsRTP*, *OsMAPK11*, *OsPAL2;3*, and *OsKSL4* by the OsMYB57 transcription factor. Chromatin DNA fragments bound to *Os*MYB57 were enriched again using an antibody to OsMYB57, and peaks 25 and 39 and 200 bp of the promoter region of *OsPAL2;3* and *OsKSL4* were used as templates to design the primers for ChIP-qPCR. Anti-IgG was used as a control.

Transcription factors function by binding to the promoter region of genes to regulate gene expression. In this study, the chromatin DNA that bound OsMYB57 was obtained using ChIP. From sequencing and alignment, a total of 75 peaks were obtained (Supplementary Dataset S2). Fifteen motifs were predicted from these peaks (Supplementary Table S4), which were significantly enriched by OsMYB57 in comparison with the anti-IgG control. On the basis of the annotation, it was shown that OsMYB57 functions in the regulation of the promoter region of genes including *retrotransposon protein* (OsRTP, LOC_Os02g23820), mitogen-activated protein kinase *11* (OsMAPK11, LOC_Os06g26340), and *OsWRKY66* (LOC_Os02g47060) (Table 1). For LOC_Os02g23820, peak 25 was annotated in the promoter region of the gene, while there were three peaks (peaks 39, 40, and 41) annotated in the promoter region of LOC_Os06g26340. These results indicate that OsMYB57 plays a role in the regulation of *OsMAPK11*, *OsRTP*, and *OsWRKY66* expression. Further prediction of the motifs from peaks 26, 39, 40, and 41 showed that 10 motifs were included in the peak (Fig. 3A), and these predicted motifs indicated the binding sites of OsMYB57 on the transcription regulation region of the promoter.

**Table 1.** Genes transcriptionally regulated by *Os*MYB57

Gene ID	Nearest gene transcriptional start site	Description	*P* value	Fold enrichment	Q value	Gene feature
*LOC_Os02g23820*	Retrotransposon protein, putative, unclassified, expressed	peak 25	1.36E-08	3.28557	0.000135	Intergenic
*LOC_Os02g47060*	Superfamily of TFs having WRKY and zinc finger domains, expressed (*OsWRKY66*)	peak 26	1.96E-46	1.92116	4.83E-41	Intergenic
*LOC_Os05g51990*	Retrotransposon protein, putative, unclassified, zinc finger, CCHC-type	peak 33	2.38E-23	1.93535	1.23E-18	Intergenic
*LOC_Os06g01090*	Retrotransposon protein, putative, Ty3-gypsy subclass, expressed	peak 36	1.28E-76	2.20759	1.04E-70	Intergenic
*LOC_Os06g26340*	CGMC_MAPKCMGC_2.10: CGMC includes CDA, MAPK, GSK3, and CLKC kinases, expressed (*OsMAPK11*)	peak 39	2.35E-25	1.54482	1.47E-20	Intergenic
		peak 40	7.24E-07	1.36267	0.004829	Intergenic
		peak 41	1.13E-05	1.41505	0.043187	Intergenic
*LOC_Os11g20790*	Adenylate kinase, putative, expressed	peak 5	4.48E-07	1.78519	0.003171	Intergenic
		peak 6	7.53E-06	1.80837	0.032164	Intergenic

### Enrichment of the promoter fragment by OsMYB57

ChIP-qPCR was conducted to determine the fold enrichment on the gene promoter regions by OsMYB57 and to validate and provide further insight into the efficiency of *Os*MYB57 in combination with the promoter of *OsRTP* (LOC_Os02g23820) *OsMAPK11* (LOC_Os06g26340). The promoters *OsPAL2;3* (LOC_Os02g41670) and *OsKSL4* (LOC_Os04g10060), which are involved in phenolic acid and momilactone B synthesis, were also evaluated using ChIP-qPCR. The enrichment of the promoter region on *OsRTP* (peak 25), *OsMAPK11* (peak 39), *OsPAL2;3*, and *OsKSL4* increased by 10.66-, 3.60-, 1.86-, and 2.00-fold, respectively (Fig. 3B). These findings validate the transcriptional regulatory capacity of OsMYB57 on *OsRTP* and *OsMAPK11*. As the enrichment on *OsPAL2;3* and *OsKSL4* was no more than 2-fold, OsMYB57 showed no direct regulation of these promoter regions.

### Interactions between OsMAPK11 and OsPAL2;3

**Fig. 4.**
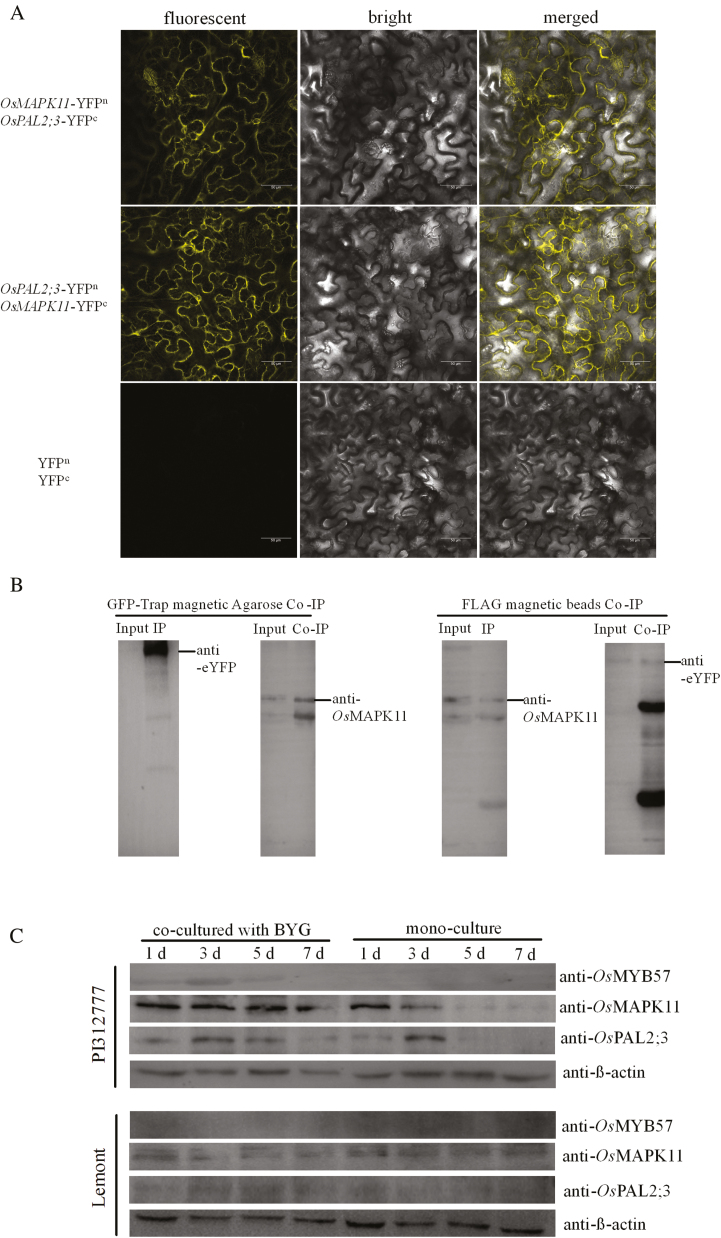
BiFC and Co-IP validation of the interaction between OsMAPK11 and *Os*PAL2;3. (A) *OsMAPK11* and *OsPAL2;3* were fused with the N-terminal domain and the C-terminal domain of YFP. The two genes with different YFP domains were then co-expressed in the leaves of *Nicotiana benthamiana* for 48 h, and YFP fluorescence was detected using laser confocal microscopy under an excitation wavelength of 488 nm. Bar=50 μm. (B) Validation by Co-IP of the interaction between OsMAPK11 and OsPAL2;3. Recombinant vectors of *OsMAPK11* fused with FLAG and *OsPAL2;3* fused with eYFP were constructed and transformed into tobacco leaves to co-express the two proteins. The total fraction of soluble proteins from the infected leaves was harvested and extracted. Proteins that interacted with OsPAL2;3 or OsMAPK11 were co-precipitated using GFP-Trap magnetic agarose and anti-FLAG M2 magnetic beads and separated by SDS-PAGE. Antibodies to eYFP and OsMAPK11 were used to detect the interaction between OsPAL2;3 and OsMAPK11. (C) Expression of OsMYB57, OsMAPK11, and OsPAL2;3 in the allelopathic rice PI312777 and the non-allelopathic rice Lemont. PI312777 and Lemont were co-cultured with barnyardgrass (BYG) at a ratio of 1:1 for 1, 3, 5, and 7 days; mono-cultured PI312777 and Lemont were used as controls. The expression of OsMYB57, OsMAPK11, and OsPAL2;3 protein was detected in each rice line; the expression of β-actin was used as a reference.

To investigate whether OsMAPK11 interacted with OsPAL2;3 to regulate phenolic acid synthesis, bimolecular fluorescence complementation (BiFC) was conducted. Based on the fluorescence signal observed by laser confocal microscopy in tobacco leaf cells when OsMAPK11 was fused with either the N-terminal domain or the C-terminal domain of YFP, it was determined that OsMAPK11 interacted with OsPAL2;3 in the cytoplasm. In contrast, no fluorescence signal was observed from the control groups (Fig. 4A). Co-immunoprecipitation (Co-IP) was simultaneously conducted to validate the interaction of OsMAPK11 with OsPAL2;3. According to a western blot analysis, the proteins collected by FLAG beads and then immunostained with anti-eYFP showed positive bands in the film, indicating positive interactions between OsMAPK11 and OsPAL2;3. Similarly, the proteins collected by GFP-Trap beads were detected using anti-OsMAPK11 antibody; the results of Co-IP and western blotting therefore further validated the positive interaction between OsMAPK11 and OsPAL2;3 (Fig. 4B). Taken together, the results of BiFC and Co-IP indicated that OsMAPK11 interacted with OsPAL2;3 *in vivo*.

### Expression of *Os*MYB57, *Os*MAPK11, and *Os*PAL2;3 in PI312777 and Lemont rice

To investigate how OsMYB57, OsMAPK11, and OsPAL2;3 were correlated with allelopathic regulation, the expression of these three proteins in the allelopathic rice PI312777 and the non-allelopathic rice Lemont co-cultured at a ratio of 1:1 with barnyardgrass and in mono-culture was evaluated. OsMYB57 was expressed in PI312777 co-cultured with barnyardgrass but was hardly detected from mono-cultured PI312777, or from either co-cultured or mono-cultured Lemont. OsMAPK11 and OsPAL2;3 were also expressed to a much higher level in PI312777 co-cultured with barnyardgrass compared with mono-cultured PI312777, indicating that the expression of these proteins was induced by the presence of barnyardgrass. By contrast, little expression was observed in either co-cultured or mono-cultured Lemont (Fig. 4C).

### Transcriptional activity of *OsPAL2;3* in PI312777 and Lemont

**Fig. 5.**
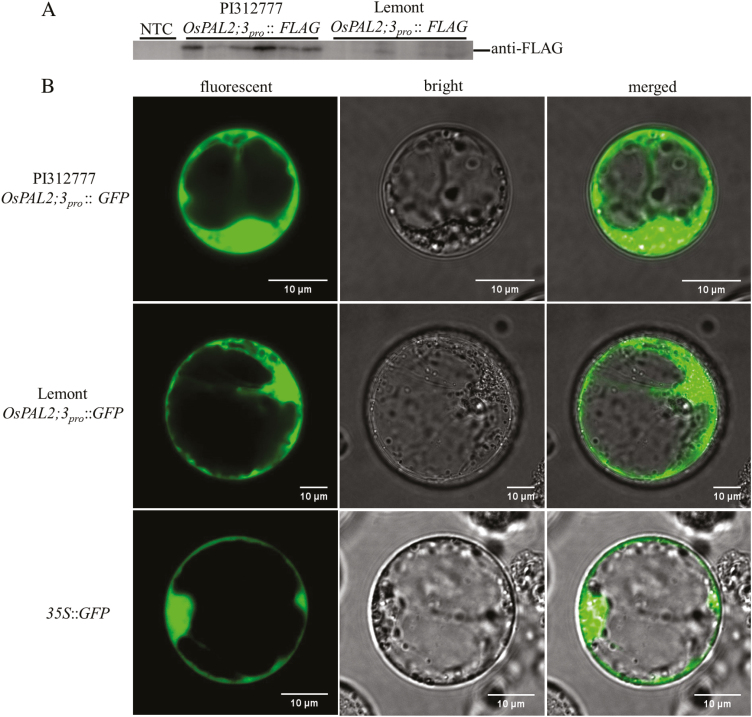
Differences in the transcriptional activity of the *OsPAL2;3* gene promoter from PI312777 and Lemont. (A) To compare the transcriptional activity of the promoter of *OsPAL2;3* from PI312777 and Lemont, the promoter region of *OsPAL2;3* was cloned from PI312777 and Lemont and fused with a FLAG tag and GFP to construct recombinant vectors, which were transformed into *Agrobacterium* EHA105 and then injected into tobacco leaves. (B) The two recombinant vectors were also transformed into rice protoplasts and the GFP fluorescence intensity was detected by laser scanning confocal microscopy.

As the first key enzyme in the phenylpropanoid pathway, OsPAL2;3 plays a vital role in the regulation of phenolic acid synthesis. We therefore compared the transcriptional activity of OsPAL2;3 in the allelopathic rice PI312777 and the non-allelopathic rice Lemont. The detection of FLAG- and GFP-fusion protein expression via western blotting and the observation of GFP fluorescence, which was driven by the *OsPAL2;3* gene promoter from PI312777 and Lemont, via laser scanning confocal microscopy were used to detect the transcriptional activity of OsPAL2;3. There were higher levels of FLAG- and GFP-fused protein expression in leaves transformed with recombinant vector containing the *OsPAL2;3* gene promoter from PI312777 compared with those containing the *OsPAL2;3* gene promoter from Lemont (Fig. 5A). There was also a higher intensity of GFP fluorescence in rice protoplasts transformed with the recombinant vector containing the *OsPAL2;3* gene promoter from PI312777 (Fig. 5B), which provides further evidence of the higher transcriptional activity of OsPAL2;3 from PI312777 compared with Lemont.

### Transcription factors binding to the promoter of *OsPAL2;3* and their gene expression levels

**Fig. 6.**
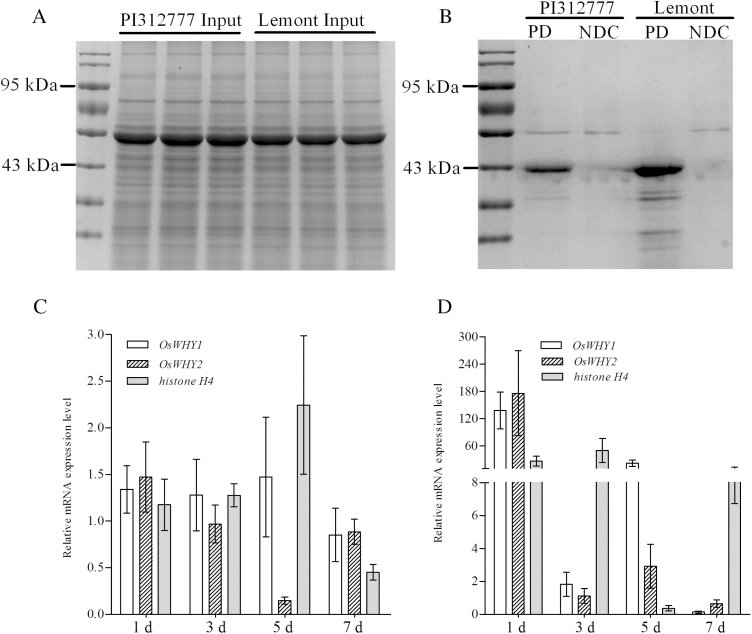
Proteins binding to the promoter of *OsPAL2;3* from PI312777 and Lemont and their input proteins. (A) The total fraction of soluble proteins from the leaves of PI312777 and Lemont was extracted and incubated with the biotin-labelled promoter of *OsPAL2;3*. (B) The proteins that bound to the promoter were co-precipitated using streptavidin-coupled Dynabeads to fish for the biotin-labelled *OsPAL2;3* promoter (PD). NDC, Proteins from Dynabeads with the incubation and fishing process. (C, D) Gene expression levels of *OsWHY* and *histone H4* in PI312777 (C) and Lemont (D). PI312777 and Lemont were co-cultured with barnyardgrass for 1, 3, 5, and 7 days; mono-cultured rice was used as a control. The gene expression of two *OsWHY*s (LOC_Os02g06370 and LOC_Os06g05350) and of *histone H4* (LOC_Os07g36500) was detected in the leaves of PI312777 and Lemont. The gene expression of *β-actin* was used as a reference, and the relative expression level was calculated according to the formula 2^–△△ct^.

As different levels of transcriptional activity of OsPAL2;3 were detected between PI312777 and Lemont, the binding of transcription factors to the promoter region of *OsPAL2;3* was investigated using the DNA pulldown method. Among the proteins identified, several with DNA-binding capacity were obtained, including histones H1 and H4. Transcriptional regulators were also identified from the promoters of both PI312777 and Lemont. In contrast, two Whirly transcription factor domain-containing proteins (*Os*WHY; LOC_Os02g06370 and LOC_Os06g05350) were identified from the *OsPAL2;3* promoter of Lemont but not from PI312777 (Fig. 6A, B; Table 2).

**Table 2.** Proteins binding on the *OsPAL2;3* gene promoter from PI312777 and Lemont

Accession	Description	Sum PEP score	Coverage	Peptides	PSMs	Unique peptides
**Lemont**						
LOC_Os06g05350.1	Whirly transcription factor domain-containing protein, expressed	44.334	50.36765	14	28	14
LOC_Os02g06370.1	Whirly transcription factor domain-containing protein, expressed	28.059	36.40351	6	14	6
LOC_Os04g42320.1	AT hook motif family protein, expressed	14.505	9.456265	9	13	9
LOC_Os05g51850.1	AT hook-containing DNA-binding protein, putative, expressed	8.184	15.24164	6	7	6
LOC_Os07g08710.1	AT hook-containing DNA-binding protein, putative, expressed	8.048	17.26619	6	9	6
LOC_Os04g58730.1	AT hook motif domain-containing protein, expressed	11.839	25.05967	5	9	5
LOC_Os08g40150.1	AT hook motif domain-containing protein, expressed	14.021	22.0339	4	6	3
LOC_Os02g31160.1	Transcription factor, putative, expressed	10.902	13.63636	4	4	4
LOC_Os09g27850.1	Transcription regulator, putative, expressed	5.362	6.136364	4	4	4
LOC_Os06g04020.1	Histone H1, putative, expressed	3.708	11.66667	3	3	3
LOC_Os07g36500.1	Histone H4, putative, expressed	4.883	21.35922	2	2	2
**PI312777**						
LOC_Os06g04020.1	Histone H1, putative, expressed	4.945	20.41667	4	4	4
LOC_Os07g36500.1	Histone H4, putative, expressed	3.038	19.41748	2	2	2
LOC_Os07g08710.1	AT hook-containing DNA-binding protein, putative, expressed	17.578	19.42446	4	18	4
LOC_Os04g58730.1	AT hook motif domain-containing protein, expressed	11.217	10.02387	3	5	3
LOC_Os04g42320.1	AT hook motif family protein, expressed	4.684	3.309693	3	3	3
LOC_Os09g27850.1	Transcription regulator, putative, expressed	3.897	4.545455	3	3	3
LOC_Os08g40150.1	AT hook motif domain-containing protein, expressed	5.682	13.27684	2	2	2

### Gene expression levels of *OsWHY* and *histone H4* in PI312777 and Lemont

To confirm the role of OsWHY (LOC_Os02g06370 and LOC_Os06g05350) and histone H4 in the regulation of promoter activity, the gene expression levels of these transcriptional regulators were detected in PI312777 and Lemont co-cultured with barnyardgrass for 1, 3, 5, and 7 days and compared with their respective control groups. Barnyardgrass induced substantially increased expression of *OsWHY1* (LOC_Os06g05350), *OsWHY2* (LOC_Os02g06370), and *histone H4* (LOC_Os07g36500) in Lemont, while these genes showed little up- or down-regulation in PI312777 (Fig. 6C, D). As the *OsPAL2;3* promoter was more active in PI312777 than in Lemont, the three DNA-binding proteins were considered to negatively regulate the transcription of *OsPAL2;3*.

## Discussion

The application of crop allelopathic traits to control weeds in farmland has recently received more attention and is recognized as a viable option for sustainable and eco-friendly agriculture (Jabran *et al.*, 2015; Aslam *et al.*, 2017; Mwendwa *et al.*, 2018; Chung *et al.*, 2018; Farooq *et al.*, 2019; Macías *et al.*, 2019). Allelopathy relies on the synthesis and secretion of allelochemical compounds by donor plants. Most of these compounds are categorized as secondary metabolites (Lotina-Hennsen *et al.*, 2006). The synthesis of the metabolites is controlled by the expression of structural genes, such as *OsPAL*, *OsKSL4*, and *OsCPS4* in rice. These metabolites are involved in pathways that ultimately determine the weed-suppression capacity of rice (Xu *et al.*, 2012; Fang *et al.*, 2013). Some special transcription factors, that is, MYB proteins, are also able to modulate the intensity of the phenylpropanoid pathway (Borevitz *et al.*, 2000; Liu *et al.*, 2015*a*; Deluc *et al.*, 2006); however, few studies have investigated the correlations among transcription factors and allelopathy traits.

The findings of the present study indicate that the transcription factor OsMYB57 functions in the regulation of rice allelopathy, because the allelopathic inhibitory ratios against barnyardgrass increased with increasing OsMYB57 expression in rice (Fig. 1A–C). Rice MYB contains at least 185 multi-gene members (Chen *et al.*, 2006; Katiyar *et al.*, 2012, and OsMYB57 belongs to the R2R3 subfamily. The R2R3-MYB subfamily has been shown to participate in the regulation of phenylpropanoid metabolism (Liu *et al.*, 2015*a*). Our study showed that the expression of genes involved in phenylpropanoid metabolism, including *OsPAL*, *OsC4H*, *OsOMT*, and *OsCCA*, was up-regulated in rice with enhanced OsMYB57, in contrast to the gene expression levels of *OsKSL4*, *OsMAS*, and *OsCPS4*, which are involved in the momilactone pathway (Fig. 1D). OsMYB57 likely played a positive role in the regulation of genes involved in phenylpropanoid metabolism. Accordingly, l-phenylalanine, which is the precursor substance for phenylpropanoid metabolism, accumulated in the OsMYB57_VP64_ transgenic line (Supplementary Table S2).

Transcription factors are well known to modulate the rate of gene transcription by binding to the DNA of promoter or enhancer regions of specific genes, and ChIP is a powerful technology to obtain the DNA region regulated by transcription factors (Carey *et al.*, 2009; Nützmann and Osbourn, 2015; Jarillo *et al.*, 2018). In this study, a combination of ChIP-seq and ChIP-qPCR revealed that OsMYB57 transcriptionally regulates the expression of the genes *OsMAPK11*, *retrotransposon protein*, and *OsWRKY66* (Table 1). The MAPK cascade is a highly conserved family of serine/threonine protein kinases involved in a variety of fundamental cellular processes, such as proliferation, differentiation, motility, stress response, apoptosis, and survival, and it is also a signal factor that amplifies the signal and mediates a broad range of biological processes (Nakagami *et al.*, 2005; Pedley and Martin, 2005). MAPK plays a vital role in transducing extracellular stimuli into intracellular responses in eukaryotic organisms, and 17 gene members have been identified from the rice genome (Rohila and Yang, 2007). These multi-gene members help to control rice grain size and weight, and the response to biotic and abiotic stresses (Jeong *et al.*, 2006; Rohila and Yang, 2007; Liu *et al.*, 2015*b*; Xu *et al.*, 2018).

In the response of rice to weeds, OsMAPK11 was predicted to be involved in the regulation of rice allelopathy against barnyardgrass through its interaction with OsPAL2;3 in vivo. The co-expression of OsMAPK11 and OsPAL2;3, which were respectively fused with the N-terminal or the C-terminal domain of eYFP, resulted in observable yellow fluorescence in the cytoplasm of *N. benthamiana* leaf cells. The positive interaction of these two proteins was also validated by Co-IP (Fig. 4A, B). The expression levels of OsMAPK11 and OsPAL2;3 protein were higher in the allelopathic rice PI312777 than in the non-allelopathic Lemont rice (Fig. 4C), while the allelopathic inhibitory ratios of PI312777 against barnyardgrass were higher than those of Lemont (Song *et al.*, 2008; He *et al.*, 2012*a*, *b*; Fang *et al.*, 2015; Zhang *et al.*, 2018, 2019). Because *PAL* is a multi-gene family in plants, *OsPAL2;3* is one of four gene members located on chromosome 2 (*OsPAL2;1*, *OsPAL2;2*, *OsPAL2;3*, and *OsPAL2;4*), and stronger transcriptional activity of *OsPAL2;3* was observed in PI312777 than in Lemont (Fig. 5A, B). Our previous studies showed that silencing the expression of *OsPAL* results in weakened allelopathic activity from the donor rice to the barnyardgrass (Fang *et al.*, 2013). The high activity of *OsPAL2;3* in the allelopathic rice was considered to contribute mainly to the increased synthesis of phenolic acid in rice plants. The transcriptional activity of genes relied on their transcription factors, while the interacting proteins acted to activate the enzyme capacity. In this study, the differences in transcriptional activity of *OsPAL2;3* from PI312777 or Lemont depended on the different types of proteins binding to the promoter region of *OsPAL2;3*. Among these proteins, OsWhirly transcription factors (LOC_Os06g05350 and LOC_Os02g06370) were identified only from the promoter of Lemont (Table 2). These proteins were considered to negatively regulate the transcription of *OsPAL2;3* because the expression levels of the genes encoding these two proteins were greatly induced in Lemont, while little change was observed in PI312777 (Fig. 6C, D). The Whirly transcription factors from Arabidopsis and potato play roles in the regulation of defence gene expression (Desveaux *et al.*, 2005), and Whirly was considered a negative regulator for the control of the rice-induced hypersensitive response (Yao *et al.*, 2008). The hypersensitive response was detectably induced in the *OsWHY*-RNAi transformed rice cells after inoculation with a non-host pathogen bacterium (Yao *et al.*, 2008). Additionally, the histone H4 that was identified among the promoter-binding proteins from both PI312777 and Lemont also showed higher expression in Lemont than in PI312777 (Fig. 6C, D; Table 2). The deacetylation of histones is often correlated with transcriptional repression (Liu *et al.*, 2014; Zhao *et al.*, 2015). High expression of histone H4 has a negative role in the regulation of *OsPAL2;3* gene expression, which represses phenolic acid synthesis in rice.

Phenolic acids that are secreted from the roots of allelopathic rice interfere with the growth of weeds (i.e. barnyardgrass) and interact with the soil microorganisms in the rhizosphere (Fang *et al.*, 2013). Our previous studies indicated that phenolic acids, including FA, CA, and HA, induce the growth and proliferation of bacteria in the culture medium of rice/weed systems, which promote weed-suppression capacity on barnyardgrass. Some species, for example, the myxobacterial species *M. xanthus*, can be induced by FA, and in a previous study the cooperation of FA and *M. xanthus* led to the highest allelopathic inhibition of barnyardgrass compared with treatment with either FA or *M. xanthus* (Fang *et al.*, 2015). The current study demonstrated that increasing the gene expression of OsMYB57 resulted in a higher population of myxobacteria and, indeed, an increase in the entire bacterial population in the rhizospheric soil (Fig. 2A). By contrast, the population of fungi was only slightly increased in OsMYB57_VP64_ rhizospheric soil (Fig. 2A). The *Bacillus* spp., *Pseudomonas* spp., and *S. viridobrunneus* isolated from rhizospheric soil showed allelopathic potential against the growth of barnyardgrass (Fig. 2B). These micro-organisms, which can be induced by FA, CA, and HA (Fig. 2C, D), are considered to have positive roles in the process of inhibition of weed growth by rice. Increasing OsMYB57 expression in rice also changed the rhizospheric community of micro-organisms, and these micro-organisms can act as a link between phenolic allelochemicals and their bio-activity in the soil.

**Fig. 7.**
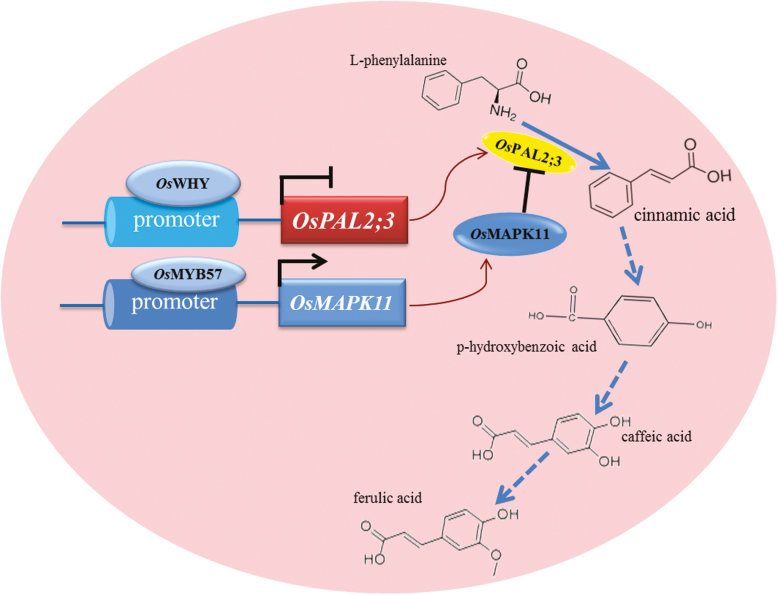
Schematic summary of the role of *Os*MYB57 in the transcriptional regulation of *Os*MAPK11, which interacts with *Os*PAL2;3 from the phenylpropanoid pathway. *Os*WHY represses the transcription of *OsPAL2;3*.

In conclusion, OsMYB57 acts as a positive transcription factor in the regulation of *OsPAL2;3* gene expression, which is dependent on transcriptional regulation of the expression of *OsMAPK11*. OsMAPK11 protein then interacts with OsPAL2;3 to regulate the activity of OsPAL2;3. The allelopathic rice PI312777 had a higher transcriptional ability of *OsPAL2;3* than the non-allelopathic rice Lemont. OsWHY and histone H4 appear to be negative factors that regulate *OsPAL2;3* transcripts (Fig. 7). These findings may provide researchers with more options for the improvement of rice allelopathic traits by genetic modification.

## Supplementary data

Supplementary data are available at *JXB* online.

Table S1. Primers used in this study.

Table S2. The relative contents of secondary metabolites detected from the root tissue of OsMYB57_VP64_ and Kitaake.

Table S3. High allelopathic microbes isolated from the rhizospheric soil of OsMYB57_VP64_ and Kitaake.

Table S4. Motifs from the sequence of peaks.

Fig. S1. The allelopathic inhibitory ratios to barnyardgrass from specific microbial strains isolated from the rhizospheric soil of Kitaake and OsMYB57_VP64_.

Dataset S1. Sequence of 16srDNA or ITS from the specific microbial strains with allelopathic inhibitory ratios to barnyardgrass.

Dataset S2. Sequence of peaks from ChIP-seq.

erz540_suppl_Supplementary_Datasets_S1-S2

erz540_suppl_Supplementary_Figure_S1

erz540_suppl_Supplementary_Table_S1-S4

erz540_suppl_Supplementary_Legends
