# Supplementary material for: MYB57 transcriptionally regulates MAPK11 to interact with PAL2;3 and modulate rice allelopathy
Source: J Exp Bot. 2019 Dec 7;71(6):2127–41. doi: 10.1093/jxb/erz540 (PMC7242072; doi:10.1093/jxb/erz540)
Supplement: erz540_suppl_Supplementary_Datasets_S1-S2 [file erz540_suppl_supplementary_datasets_s1-s2.pdf]

Supplementary dataset S1. Sequence of 16srDNA or ITS from the specific microbial strains with allelopathic inhibitory ratios to barnyardgrass

Microbial ID: V2, ***Pseudomonas*** **spp. YXE3-18, 16srDNA**

>ACACATGCAGTCGAGCGGTAGAGAGAAGCTTGCTTCTCTTGAGAGCGGCGGACGGG
TGAGTAAAGCCTAGGAATCTGCCTGGTAGTGGGGGATAACGTTCGGAAACGGACGCTA
ATACCGCATACGTCCTACGGGAGAAAGCAGGGGACCTTCGGGCCTTGCGCTATCAGAT
GAGCCTAGGTCGGATTAGCTAGTTGGTGAGGTAATGGCTCACCAAGGCGACGATCCGT
AACTGGTCTGAGAGGATGATCAGTCACACTGGAACTGAGACACGGTCCAGACTCCTAC
GGGAGGCAGCAGTGGGGAATATTGGACAATGGGCGAAAGCCTGATCCAGCCATGCCG
CGTGTGTGAAGAAGGTCTTCGGATTGTAAAGCACTTTAAGTTGGGAGGAAGGGCATTA
ACCTAATACGTTAGTGTTTTGACGTTACCGACAGAATAAGCACCGGCTAACTCTGTGCC
AGCAGCCGCGGTAATACAGAGGGTGCAAGCGTTAATCGGAATTACTGGGCGTAAAGCG
CGCGTAGGTGGTTTGTTAAGTTGGATGTGAAATCCCCGGGCTCAACCTGGGAACTGCA
TTCAAAACTGACTGACTAGAGTATGGTAGAGGGTGGTGGAATTTCCTGTGTAGCGGTG
AAATGCGTAGATATAGGAAGGAACACCAGTGGCGAAGGCGACCACCTGGACTAATACT
GACACTGAGGTGCGAAAGCGTGGGGAGCAAACAGGATTAGATACCCTGGTAGTCCAC
GCCGTAAACGATGTCAACTAGCCGTTGGGAGCCTTGAGCTCTTAGTGGCGCAGCTAAC
GCATTAAGTTGACCGCCTGGGGAGTACGGCCGCAAGGTTAAAACTCAAATGAATTGAC
GGGGGCCCGCACAAGCGGTGGAGCATGTGGTTTAATTCGAAGCAACGCGAAGAACCT
TACCAGGCCTTGACATCCAATGAACTTTCTAGAGATAGATTGGTGCCTTCGGGAACATT
GAGACAGGTGCTGCATGGCTGTCGTCAGCTCGTGTCGTGAGATGTTGGGTTAAGTCCC
GTAACGAGCGCAACCCTTGTCCTTAGTTACCAGCACGTAATGGTGGGCACTCTAAGGA
GACTGCCGGTGACAAACCGGAGGAAGGTGGGGATGACGTCAAGTCATCATGGCCCTT
ACGGCCTGGGCTACACACGTGCTACAATGGTCGGTACAGAGGGTTGCCAAACCGCGA
GGTGGAGCTAATCCCACAAAACCGATCGTAGTCCGGATCGCAGTCTGCAACTCGACTG
CGTGAAGTCGGAATCGCTAGTAATCGCGAATCAGAATGTCGCGGTGAATACGTTCCCG
GGCCTTGTACACACCGCCCGTCACACCATGGGAGTGGGTTGCACCAGAAGTAGCTAGT
CTAACCTTCGGGAGGACGGTAC

Microbial ID: V11, ***Pseudomonas*** **spp. J3.2C5, 16srDNA**

>GCTACACATGCAGTCGAGCGGTAGAGAGAAGCTTGCTTCTCTTGAGAGCGGCGGACG
GGTGAGTAAAGCCTAGGAATCTGCCTGGTAGTGGGGGATAACGTTCGGAAACGGACG
CTAATACCGCATACGTCCTACGGGAGAAAGCAGGGGACCTTCGGGCCTTGCGCTATCA
GATGAGCCTAGGTCGGATTAGCTAGTTGGTGAGGTAATGGCTCACCAAGGCGACGATC
CGTAACTGGTCTGAGAGGATGATCAGTCACACTGGAACTGAGACACGGTCCAGACTCC
TACGGGAGGCAGCAGTGGGGAATATTGGACAATGGGCGAAAGCCTGATCCAGCCATGC
CGCGTGTGTGAAGAAGGTCTTCGGATTGTAAAGCACTTTAAGTTGGGAGGAAGGGCAT
TAACCTAATACGTTAGTGTTTTGACGTTACCGACAGAATAAGCACCGGCTAACTCTGTG
CCAGCAGCCGCGGTAATACAGAGGGTGCAAGCGTTAATCGGAATTACTGGGCGTAAAG
CGCGCGTAGGTGGTTTGTTAAGTTGGATGTGAAATCCCCGGGCTCAACCTGGGAACTG

CATTCAAAACTGACTGACTAGAGTATGGTAGAGGGTGGTGGAATTTCCTGTGTAGCGG
TGAAATGCGTAGATATAGGAAGGAACACCAGTGGCGAAGGCGACCACCTGGACTAATA
CTGACACTGAGGTGCGAAAGCGTGGGGAGCAAACAGGATTAGATACCCTGGTAGTCC
ACGCCGTAAACGATGTCAACTAGCCGTTGGGAGCCTTGAGCTCTTAGTGGCGCAGCTA
ACGCATTAAGTTGACCGCCTGGGGAGTACGGCCGCAAGGTTAAAACTCAAATGAATTG
ACGGGGGCCCGCACAAGCGGTGGAGCATGTGGTTTAATTCGAAGCAACGCGAAGAAC
CTTACCAGGCCTTGACATCCAATGAACTTTCTAGAGATAGATTGGTGCCTTCGGGAACA
TTGAGACAGGTGCTGCATGGCTGTCGTCAGCTCGTGTCGTGAGATGTTGGGTTAAGTC
CCGTAACGAGCGCAACCCTTGTCCTTAGTTACCAGCACGTAATGGTGGGCACTCTAAG
GAGACTGCCGGTGACAAACCGGAGGAAGGTGGGGATGACGTCAAGTCATCATGGCCC
TTACGGCCTGGGCTACACACGTGCTACAATGGTCGGTACAGAGGGTTGCCAAACCGCG
AGGTGGAGCTAATCCCACAAAACCGATCGTAGTCCGGATCGCAGTCTGCAACTCGACT
GCGTGAAGTCGGAATCGCTAGTAATCGCGAATCAGAATGTCGCGGTGAATACGTTCCC
GGGCCTTGTACACACCGCCCGTCACACCATGGGAGTGGGTTGCACCAGAAGTAGCTAG
TCTAACCTTCGGGAGGACGGTACC

Microbial ID:V16 , *Penicillium aculeatum* strain H23, ITS

>TCCTCCGCTTATTGATATGCTTAAGTTCAGCGGGTAACTCCTACCTGATCCGAGGTCAA
CCGTGGTAAAAAAAACATGGTGGTGACCAACCCCCGCAGGTCCCTCCCGAGCGAGTG
ACAGAGCCCCATACGCTCGAGGACCAGACGGACGTCGCCGCTGCCTTTCGGGCAGGT
CCCCGGGGGGACCACACCCAACACACAAGCCGTGCTTGAGGGCAGAAATGACGCTCG
GACAGGCATGCCCCCCGGAATGCCAGGGGGCGCAATGTGCGTTCAAAGATTCGATGAT
TCACGGAATTCTGCAATTCACATTACTTATCGCATTTCGCTGCGTTCTTCATCGATGCCG
GAACCAAGAGATCCATTGTTGAAAGTTTTGACAATTTTCATAGTACTCAGACAGCCCAT
CTTCATCAGGGTTCACAGAGCGCTTCGGCGGGCGCGGGCCCGGGGACGTGCGTCCCC
CGGCGACCAGGTGGCCCCGGTGGGCCCGCCAAAGCAACAGGTGTATAGAGACAAGGG
TGGGAGGTTGGGCCGCGAGGGCCCGCACTCGGTAATGATCCTTCCGCAGGTTCACCTA
CGGAAACCTTGTTACGACTTTTACTTCTCTAAATTGACCA

Microbial ID: V29, *Penicillium rubidurum* isolate CY249, ITS

>CTTGGTCATTTTAGAGAAGTAAAAGTCGTAACAAGGTTTCCGTAGGTGAACCTGCGG
AAGGATCATTACCGAGTGAGGGCCCTCTGGGTCCAACCTCCCACCCGTGTTTATCGTAC
CTTGTTGCTTCGGCGGGCCCGCCGCAAGGCCGCCGGGGGGCTTCCGTCCCCGGGCCC
GCGCCCGCCGAAGACACCTGTGAACGCTGTATGAAGATTGCAGTCTGAGCGAAAAGC
TAAATTTATTAAAACTTTCAACAACGGATCTCTTGGTTCCGGCATCGATGAAGAACGCA
GCGAAATGCGATAAGTAATGTGAATTGCAGAATTCAGTGAATCATCGAGTCTTTGAACG
CACATTGCGCCCCCTGGTATTCCGGGGGGCATGCCTGTCCGAGCGTCATTGCTGCCCTC
AAGCACGGCTTGTGTGTTGGGCCCTCGTCCCCCGGGACGGGCCCGAAAGGCAGCGGC
GGCACCGCGTCCGGTCCTCGAGCGTATGGGGCTTCGTCACCCGCTCTGTAGGCCCGGC
CGGCGCCTGCCGACACCATCAATCTTTTTTCCAGGTTGACCTCGGATCAGGTAGGGATA
CCCGCTGAACTTAAGCATATCAATAAGCGGAGGAA

Microbial ID: V32, *Streptomyces Viridobrunneus* STRAIN SCPE-09, 16SRDNA

>TGCAGTCGAACGATGAAGCCCTTCGGGGTGGATTAGTGGCGAACGGGTGAGTAACAC
GTGGGCAATCTGCCCTTCACTCTGGGACAAGCCCTGGAAACGGGGTCTAATACCGGAT
ACGACCTGCCGAGGCATCTTGGGGGGTGGAAAGCTCCGGCGGTGAAGGATGAGCCCG
CGGCCTATCAGCTTGTTGGTGGGGTAATGGCCTACCAAGGCGACGACGGGTAGCCGGC
CTGAGAGGGCGACCGGCCACACTGGGACTGAGACACGGCCCAGACTCCTACGGGAGG
CAGCAGTGGGGAATATTGCACAATGGGCGAAAGCCTGATGCAGCGACGCCGCGTGAG
GGATGACGGCCTTCGGGTTGTAAACCTCTTTCAGCAGGGAAGAAGCGCAAGTGACGG
TACCTGCAGAAGAAGCGCCGGCTAACTACGTGCCAGCAGCCGCGGTAATACGTAGGGC
GCAAGCGTTGTCCGGAATTATTGGGCGTAAAGAGCTCGTAGGCGGCTTGTCACGTCGG
GTGTGAAAGCCCGGGGCTTAACCCCGGGTCTGCATCCGATACGGGCAGGCTAGAGTGT
GGTAGGGGAGATCGGAATTCCTGGTGTAGCGGTGAAATGCGCAGATATCAGGAGGAAC
ACCGGTGGCGAAGGCGGATCTCTGGGCCATTACTGACGCTGAGGAGCGAAAGCGTGG
GGAGCGAACAGGATTAGATACCCTGGTAGTCCACGCCGTAAACGTTGGGAACTAGGTG
TTGGCGACATTCCACGTCGTCGGTGCCGCAGCTAACGCATTAAGTTCCCCGCCTGGGG
AGTACGGCCGCAAGGCTAAAACTCAAAGGAATTGACGGGGGCCCGCACAAGCAGCGG
AGCATGTGGCTTAATTCGACGCAACGCGAAGAACCTTACCAAGGCTTGACATATACCG
GAAAGCATTAGAGATAGTGCCCCCCTTGTGGTCGGTATACAGGTGGTGCATGGCTGTCG
TCAGCTCGTGTCGTGAGATGTTGGGTTAAGTCCCGCAACGAGCGCAACCCTTGTCCTG
TGTTGCCAGCATGCCCTTCGGGGTGATGGGGACTCACAGGAGACCGCCGGGGTCAAC
TCGGAGGAAGGTGGGGACGACGTCAAGTCATCATGCCCCTTATGTCTTGGGCTGCACA
CGTGCTACAATGGCCGGTACAAAGAGCTGCGATGCCGTGAGGCGGAGCGAATCTCAA
AAAGCCGGTCTCAGTTCGGATTGGGGTCTGCAACTCGACCCCATGAAGTCGGAGTTGC
TAGTAATCGCAGATCAGCATTGCTGCGGTGAATACGTTCCCGGGCCTTGTACACACCGC
CCGTCACGTCACGAAAGTCGGTAACACCCGAAGCCGGTGGCCCAACCCCTTGTGGGA
GGGAG

Microbial ID: K15 *Streptomyces* spp. FXJ1.430, 16srDNA

>GGACCGGGGGGCTGCTCACCTGCAAGTCGAACGATGAACCACTTCGGTGGGGATTAG
TGGCGAACGGGTGAGTAACACGTGGGCAATCTGCCCTGCACTCTGGGACAAGCCCTG
GAAACGGGGTCTAATACCGGATATGAGCCGCGCCCGCATGTGCCTGGCTGTAAAGCTC
CGGCGGTGCAGGATGAGCCCGCGGCCTATCAGCTTGTTGGTGAGGTAACGGCTCACCA
AGGCGACGACGGGTAGCCGGCCTGAGAGGGCGACCGGCCACACTGGGACTGAGACA
CGGCCCAGACTCCTACGGGAGGCAGCAGTGGGGAATATTGCACAATGGGCGAAAGCC
TGATGCAGCGACGCCGCGTGAGGGATGACGGCCTTCGGGTTGTAAACCTCTTTCAGCA
GGGAAGAAGCGAAAGTGACGGTACCTGCAGAAGAAGCGCCGGCTAACTACGTGCCAG
CAGCCGCGGTAATACGTAGGGCGCAAGCGTTGTCCGGAATTATTGGGCGTAAAGAGCT
CGTAGGCGGCTTGTCGCGTCGGTTGTGAAAGCCCGGGGCTTAACCCCGGGTCTGCAGT
CGATACGGGCAGGCTAGAGTTCGGTAGGGGAGATCGGAATTCCTGGTGTAGCGGTGAA
ATGCGCAGATATCAGGAGGAACACCGGTGGCGAAGGCGGATCTCTGGGCCGATACTGA
CGCTGAGGAGCGAAAGCGTGGGGAGCGAACAGGATTAGATACCCTGGTAGTCCACGC
CGTAAACGGTGGGCACTAGGTGTGGGCAACATTCCACGTTGTCCGTGCCGCAGCTAAC

GCATTAAGTGCCCCGCCTGGGGAGTACGGCCGCAAGGCTAAAACTCAAAGGAATTGA
CGGGGGCCCGCACAAGCGGCGGAGCATGTGGCTTAATTCGACGCAACGCGAAGAACC
TTACCAAGGCTTGACATACACCGGAAAACCCTGGAGACAGGGTCCCCCTTGTGGTCGG
TGTACAGGTGGTGCATGGCTGTCGTCAGCTCGTGTCGTGAGATGTTGGGTTAAGTCCC
GCAACGAGCGCAACCCTTGTCCCGTGTTGCCAGCAGGCCCTTGTGGTGCTGGGGACTC
ACGGGAGACCGCCGGGGTCAACTCGGAGGAAGGTGGGGACGACGTCAAGTCATCATG
CCCCTTATGTCTTGGGCTGCACACGTGCTACAATGGCCGGTACAATGAGCTGCGATACC
GTGAGGTGGAGCGAATCTCAAAAAGCCGGTCTCAGTTCGGATTGGGGTCTGCAACTC
GACCCCATGAAGTCGGAGTCGCTAGTAATCGCAGATCAGCATTGCTGCGGTGAATACG
TTCCCGGGCCTTGTACACACCGCCCGTCACGTCACGAAAGTCGGTAACACCCGAAGCC
GGTGGCCCAACCCCTTGTGGGAGGGAGCTTCGAAGTGACGCATCAGTCGTCTA

Supplementary dataset S2. Sequence of peaks from ChIP-seq

Peak number	Sequence
peak_1::Chr1:16893528-16893728	TTAGTACCAATATTGGCATATATTGAGCCCTCACGGGTGCGATGTTTTTTGACCGGAATGAAAAAGTTCAAAAAGCACCAAAACAAGATTTTTGGACATATTGGAGTGTATTTGGTGCGTTCGTAGCAAAAACTCACTTCGTGATTTGCGCGGCGAACTTTTGTCAATTTATGCCAATCTTGGCATATATTGAGCCCTCA
peak_2::Chr1:18734583-18734783	GCTCAAAATACCCTCGCCGACCTGGAGCGCCTGGTGCAAGATCAGGCTGGGGAGATCGCGGCCCTCCGCCTCACCAACGAGATCGGACCCGGGCAGCTCTCCGACGCCGTCGACCGGCTGGAGCGCGCGGGGCGCCGAGTCGGCATTTCTGTGCGCCGGGATAGCAAACTTCCGCCCACACAGCCAGCACTCATGCTCCG
peak_3::Chr1:26852688-26852888	GGGCCGCGAGGCCGCCGCTGGTCTCCGAGAGGCGACGCTGGTGGCGCATGAGGCCGCCTGCGCCGAGGAGGAGTCCGCGCTCCGCCTTCGCGAGGATGCACTCGCTGAGCGGGAGCGAGCTCTCGAGGAGGCCGAGGCCACGACGCAACGGCTGGCGAACAGCCTCTCCCTCCGCGAGGCGGCGCAAGAGGAGCAGGCGC
peak_4::Chr11:10606060-10606260	ACCGCTGCTGCCGATGACCGGTGCAGGCCGGGCAGCAGCGGTAGCGACGAGCGCCGTGGGTGGCGGCGCGATGGGCGCGGGTGGCGTAGGCAGCGCGACGGCCGCGGGTGGCTCAGCCGGCGTGGTGGGCTGGTGGCACTTGGCGCGGGTGGCTTAGCTGCTGCAGCAGTGCAGGGAAGCGGGCGCGCGGCAGGCAGCCA
peak_5::Chr11:12042483-12042683	ACGCCTATTTTAAGAAATGACACCCGAATGACGCCAAAGCATGTCGGATGCGATCATACCAGCACTAAAGCACCGGATCCCATCAGAACTCCGAAGTTAAGCGTGCTTGGGCGAGAGTAGTACTAGGATGGGTGACCTCCTGGGAAGTCCTCGTGTTGCATCCCTCCTTTTTGTCTCTCTCTCCCCCCTTTTGACTCGCG
peak_6::Chr11:12109639-12109839	AGCACTAAAGCACCGGATCCCATCAGAACTCCGAAGTTAAGCGTGCTTGGGCGAGAGTAGTACTAGGATGGGTGACCTCCTTGGAAGTCCTCGTGTTGCATCCCTCCTTTTTGTCTCTCTCTCCCCCCTTTTGACTCGCGCCGCTGCGTCCATCGTGTTGTGTCGCCCCTTGGGCGGCGAAGCTGGGGAGAATCGGATGT
peak_7::Chr11:12170851-12171051	ATGTCCGTGGATATATCATTTGCCTGATTCCGAGTCCGTATGAGAAAGTTACGCCTATTTTAAGAAATGACACCCGAATGACGCCAAAGCATGTCGGATGCGATCATACCAGCACTAAAGCACCGGATCCCATCAGAACTCCGAAGTTAAGCGTGCTTGGGCGAGAGTAGTACTAGGATGGGTGACCTCCTGGGAAGTCC
peak_8::Chr11:12183577-12183777	ATTGTCAAAAGTTCGCCGCGCGAATCCAGAAGTGTATTTTTGACACGAACGCACCCAATACACTCCAATATGTCCAAAAATCATGTTTTGGTGCTTTTTGAACCTTTTCTTTCCGGTCAAAAACATCGCACCCGTGTGAGCCAATTTATGCCAATATTGGCATTAATGGACAAAAGTTCGCCGCGCGAATCAAGAAGTAA
peak_9::Chr11:12188818-12189018	AGTAATTGTCAAAAGTTCGCCGCGCGAATCCAGAAGTGTATTTTTGACACGAACGCACCCAATACACTCCAATATGTCCAAAAATCATGTTTTGGTGCTTTTTGAACCTTTTCTTTCCGGTCAAAAACATCGCACCCGTGTGAGCCAATTTATGCCAATATTGGCATTAATGGACAAAAGTTCGCCGCGCGAATCAAGAA
peak_10::Chr11:12267327-12267527	AATCATGTTTTGGTGCTTTTTGAACTTTTTCATTCCGGTCAAAAACATCGCACCCGTGTGGGGCCATATTGCCATTTATGGACAAAAGTTCGCCGCGCGAATCAAGAAGTAAGTTTTTGCCACGAACGCACCCAATACACTCCAATATGTCCAAAATTCATGTTTTGGTGCTTTTTGTACCTTTTCATTCCGGTCAAAAA

peak_11::Chr11:12272457-12272657	CCAATATTGGCATTAATTGACAAAAGTTCGCCGCGCGAATCCAGATGTGTATTTTTGCCACGAACGCACCCAATACACTCCAATATGTCCAAAAATCATGTTTTGGTGCTTTTTGAACCTTTTCTTTCCGGTCAAAAGCATCGCACCCGTGTGAGCCAATATATGCCAATATTGGCATTAATGGACAAAAGTTCGCCACG
peak_12::Chr11:12278792-12278992	ACTTCTGGATTCGCGCGGCGAACTTTTGTCAATTAATGCCAATATTGGCATATATTGGCTCACACGGGTGCGATGTTTTTGACCGGAAAGAAAAGGTTCAAAAAGCACCAAAACATGATTTTTGGACATATTGGAGTGTATTGGGTGCGTTCGTGGCAAAAAGACACTTCTGGATTCGCGCGGCGAACTTTTGTCAATTA
peak_13::Chr11:12280600-12280800	TGGAGTGTATTGGGTGCGTTCTTAGCAAAAACTTACTTCTTGATTCGCGCGGCGAACTTTTGTCCATTAATGCCAATATTGCCCCACACGGGTGCGATGTTTTTGACCGGAATGAAAATGTTCAAAAAAGCACCAAAACATGATTTTTGGACATATTGTGTGCTTTCGTGGAAAAAACTTACTTCTCGATTCGCGCGGCG
peak_14::Chr11:12295938-12296138	AATATATGCCAATATTGGCATTAATTGACAAAAGCTCGCCACGCGAATCAAGAAGTAAGTTTTTGCCACGAACGCACCCAATACACTCCAATATGTCCAAAAATCATGTTTTGGTGCTTTTTGAACAGTTTCATTCCGGTCAAAAACATCGCACCCGTGTGAGCCAATATATGCCAATATTGGCATTAATTGACAAAAGC
peak_15::Chr11:12301816-12302016	AATCATGTTTTGGTGCTTTTTGTACCTTTTCTTTCCGGTCAAAAACATCGCACCCGTGTGAGCCAATATATGCCAATATTGGCATTAATTGACAAAAGTTCGCCGCGCGAATCCAGAAGTGTGTTTTTGCCACGAACGCACCCAATACACTCCAATATGTCCAAAAATCGTGTTTTGGTGCTTTTTGAACTTTTTCATTC
peak_16::Chr11:12303272-12303472	GGTGCTTTTTGAACTTTTTCAATCCGGTCAAAAACATAACACCCGTGTGGGGCAATATTGGCATTTATGGACAAAAGTTCGCCGCGCGAATCAAGAAGTAAGTTTTTGCCACGAACGCACCCAATACACTCCAATATGTCCAAAAATCATGTTTTGGTGCTTTTTGAGCTTTTTCATTCTGGTCAAAAACATCGCCCCCG
peak_17::Chr11:12315907-12316107	CCGCGCGAATCGAGAAGTAAGTTTTTTCCACGAAAGCACACAATATGTCCAAAAATCATGTTTTGGTGCTTTTTGAACATTTTCATTCCGGTCAAAAACATCGCACCCGTGTGGGGCAATATTGGCATTAATTGACAAAAGCTCGCCACGCGAATCAAGAAGTAAGTTTTTGCCACGAACGCACACAATACACTCCAATA
peak_18::Chr11:12321154-12321354	NNNNNNNNNNNNNNNNNNNNNNNNNNNNNNNNNNNNNNNCACACAATACACTCCAATATGTCCAAAAATCATGTTTTGGTGCTTTTTGAGCTTTTTCATTCCGGTCAAAAACATCGCACCCGTGTGGGCCAATATATGCCAATATTGGCATTAATTGACAAAAGTTCGCCGCGCGAATCCAGAAGTAAGTTTTTGCCACGAA
peak_19::Chr11:12322470-12322670	TGGCATTAATTGACAAAAGTTCGCCGCGCGAATCCAGATGTGTATTTTTGCCACGAACGCACCCAATACACTCCAATATGTCCAAAAATCATGTTTTGGTGCTTTTTGAACCTTTTCTTTCCGGTCAAAAGCATCGCACCCGTGTGAGCCAATATATGCCAATATTGGCATTAATGGACAAAAGTTCGCCACGCGAATCA
peak_20::Chr12:10104424-10104624	TGAATAGAATTTGCCGGTAAAGAAGTAAACCATGTAAAATCATTACCGGATAAAGATAAAGAAAATAAGCGCAATTTAAAAGTATCACTATTAGCCTCACCACATTGCGCTAAAAATTGACCTACATGCTCCCATGTGGTCTTACCATCCTCACCACTAAACTTGGTAAATTCGGGAACTCTATAATTTTTAGGAAACGA
peak_21::Chr12:11991875-11992075	TTTGGACATATTGGAGTGTGGTTGGTGCATTCGTGGAAAAAAAATCACTTCGTGATTCGCGCGGCGAACTTTTGTCAATTACTGCCAATATTGGCATAGGTGGGTGCGATGTTTTTCACCGGAATGAAAATCACTTCGTGATTCGCGCGGCGAACTTTTGTCAATTACTGCCATTATTGGCATAGGTGGGTGCGATGTTT

peak_22::Chr12:11<br>993253-11993453	CGTGGCAAAAAATCACTTCGTGATTCGCGCGGCGAACTTTTGTCAATTAATGCCA<br>ATATTGCCATAGGTGGGTGCGATGTTTTTCACCGGAATGAAAAAGTTCAAAAAG<br>CACCAAAACATGATTTTTGGACATATTGGAGTGTGTTGGGTGCATTCGTGGCAAA<br>AAATCACTTCGTGATTCGCGCGGCGAACTTTTGTCA
peak_23::Chr12:11<br>995279-11995479	TGCCATAGGTGGGTGCGATGTTTTTCACCGGAATGAAAAAGTTCAAAAAGCACC<br>AAAACATGATTTTTGGACATATTGGAGTGTGGTTGGTGCATTCGTGGAAAAAAAA<br>TCACTTCGTGATTCGCGCGGCGAACTTTTGTCAATTACTGCCAATATTGGCATAG<br>GTGGGTGCGATGTTTTTCACCGGAATGAAAATCACT
peak_24::Chr12:12<br>008746-12008946	TATTGGCAGTAATTGACAAAAGTTCGCCGCGCGAATCACGAAGTGATTTTTTGCC<br>ACGAACGCACCCAACACACTCCAATATGTCCAAAAATCATGTTTTGGTGCTTTTT<br>GAACTTTTTCATTCCGGTGAAAAACATCGCACCCACCTATGGCAATATTGGCATT<br>AATTCACAAAAGTTCGCCGCGCGAATCACGAAGTG
peak_25::Chr2:136<br>68495-13668695	GGCAAAAAGTCACCTCGTGATTCGCCCGGCGAACTTTTGTCAATTAATGCAAATA<br>TTGGCCGACACGGGTGCGATGTTTTTGACCGGAATGAAATAGTTCAAAAAGCAC<br>CAAAACATGATTTTTGGACATATTGGAGTGTATTGGGTGCGTTCGTGGCAAAAAC<br>TCGCTTCATGATTCGCTTGGCGAACTTTTGTCAATT
peak_26::Chr2:287<br>20160-28720360	ATGGGATAACATCATAGGATTCCGGTCCTATTGTGTTGGCCTTCGGGATCGGAGT<br>AATGATTAATAGGGACAGTCGGGGGCATTCGTATTTCATAGTCAGAGGTGAAATT<br>CTTGGATTTATGAAAGACGAACAACTGCGAAAGCATTTGCCAAGGATGTTTTCAT<br>TAATCAAGAACGAAAGTTGGGGGCTCGAAGACGAT
peak_27::Chr3:135<br>14647-13514847	GGCAAGCCGAGCAATGTACCCAAGTTAGTGTTTTAATTCTTAAAACTTGCTTAAA<br>CAACTAAAATGTGGAATGGTTGGCCTGGGTTGGCTTGGGACGAGCTGGGACCCA<br>GGGTCGGGTTGCCAGTTCGGTCCGGATCATCGTAGGCCTTGGGTTAAGGCAGGT<br>TCGTGTGGGTTCACGGCCTTGATTAATAACATTGTAT
peak_28::Chr3:195<br>58542-19558742	TGCGTTGATGGCAAAAACTCACTTCGCGACTCCCACGGCGAACTTTTTTGAATTA<br>ATGCCGATATTGCCACACGTGGGTGGGATGTAATATACCGGAATCAAAAAGTTCA<br>AAAAGCACCAAAACATTATTTTTGGACATATTGCAGTGTATTGGGTGCGTTTGTG<br>GCAAAAACTTACTTCGCGACTCGCGCGGTGAACTT
peak_29::Chr3:195<br>62582-19562782	GGCAAAAACTCACTTCGCGACTCCCACGGCGAACTTTTTTGAATTAATGCCGATA<br>TTGCCACACGTGGGTGGGATGTAATATACCGGAATCAAAAAGTTCAAAAAGCAC<br>CAAAACATTATTTTTGGACATATTGCAGTGTATTGGGTGCGTTTGTGGCAAAAAC<br>TTACTTCGCGACTCGCGCGGTGAACTTTTGTCAACT
peak_30::Chr3:195<br>66302-19566502	GACTCGCGCGGTGAACTTTTGTCAATTAATGCCGATATTGCCACATGTGGGTGCG<br>ATGTAATATACCAGAATCAAAAAGTTTAAAAAGCACCTAAACATGATTTTTGGAC<br>ATATTGGAGTGTATTGGGAGCATTCGTGGCAAAAACTCACTTCGTGGCATGCGCG<br>GCGAACTTTTGTCAATTAATGCTGATATTGTCACA
peak_31::Chr4:287<br>3165-2873365	TTGCATTGCCGTGATGAAGTGAACACGAGTCGTCGGCAACAGAAGCAAACTGGT<br>AGTGAAGACGCGCAGTTGTGAAGATAAAAGCACTAGTCGGCGACAGCATAACC<br>AGTCGGCGACGGAAGACGATGATGTTCATCAGTAGTGGTAGCTGTATAAACCAG<br>ATGTAGAGGCGAGGGCACTAGTCAGCAGCAGACGAGACG
peak_32::Chr4:119<br>97700-11997900	AGAAGACCACCGATCCGTGTGGTCTTGGATGGGCCGTCGCCCAAGGCAACCTCC<br>TTGACCGGGACCTCGTCGCATGTGGCAATCCGCTGCCTCGGGGCGGTGGGGGCG<br>GAGGTGCCAAGCCTCTCGTCACCACCCTCGGGCTTGGTCGCAGCAGCGAGGTG

	GTCCGCGCGCTGCTCCATGCAAGCGAGCGCGACTTTAAG
peak_33::Chr5:29855378-29855578	GGATTTGGAGAGTTGGACCCCCAGAGATTTTTCTAGAAATTACTTACCATAGGCCACGAAGTAGCGGTTGTTTTGGCGAAACGTCGAATATCTGAGGGTCCACAACTGGTTTTCAACGAGGTGGTTAGTATAGGGATGGCACATAGGACGTTAGGGTGGATAGCTTAGACGATGTCGGTGTCAAGCGCGTCGACTAACCG
peak_34::Chr6:32271-32471	TTGATAACTTTGCCTTCCGGTCATGGAATCGAGCTAGGTTTTTTTTGACGGCTGCAGAATCACAGGCCTCACCGAAGAGAGAATGGATTTGGAGAGTTGGACCCCCAGAGATTTTTCTAGAAATTACTTACCATAGGCCACGAAGCAGCGGTTGTTTCGGCGAAACGTCGAATATCTGAGGGTCCACAACTGGTTTTCAA
peak_35::Chr6:56241-56441	GTTAGGGTGGATAGCTTAGACGATGTCGGTGTCAAGCGCGTCGACTAACCGCGCCGTTAAGGTAGGCTGGCGGGCGCGACACTGTGGAATGCAGATGGAGAATTCTGTGCAAATTAAAACCTTGATAACTTTGCCTTCCGGTCATGGAATCGAGCTAGGTTTTTTTTGACGGCTGCAGAATCACGGGCCTCACCCTAGAG
peak_36::Chr6:84211-84411	ACTAACCGCGCCGTTAAGGTAGGCTGGCAGGCGCGACACTGTGGAATGCAGATGGAGAATTCTGTGCAAATTAAAACCTTGATAACTTTGCCTTCCGGTCATGGAATCGAGCTAGGTTTTTTTTGACGGCTGCAGAATCACGGGCCTCACCATAGAGAGACTGGATTTGGAGAGTTGGACCCCCAGAGATTTTTCTAGAA
peak_37::Chr6:9295235-9295435	CCCAGATCGCGCCTCCATCTTGACCATTATGACGGCGGTCGGGGCCTCAGAGGAGCAGGCTCCTAAGGGCCATGACGGCGCAGGTGGGAGCCGCCGGGGGGATCAATCTACCCCCGGAGGGGGTCGTGCTTCTGGGCCCCGCGTCGGGGGCTCGGGGAGCAACCGCCCTACCGACGCCCGGGGGAAGAGGAAACTGGGAG
peak_38::Chr6:10623476-10623676	TGCGGTTGTCGGCCCCGGTACCCGTGTATCGGCGTCGGCAGTCGTTCGCTTGTGCGCGTGGTGACCGTGTTAGTGTGTCCGCTCGGTAGCCGCGTGGTTTCCACGTGTGCAGTCCGTGTGGTGTCTAGTGGAGTCCGTTTGTCGGCCACTCGCCTCGGTTGACACACGGGATCCGCTTCCGTCGACCCGTGGACCGACTC
peak_39::Chr6:15425971-15426171	GGACATATTGGAGTGTATTGGGTGGGTTCGTGGCAAAAACTCACTTCGCGACTCATGCGGCGAACTTTTGTCAATTAATGCCGATATTGCCACACGTGGGTGCGATGTAATATACCGGAATCAAAAAGTTCAAAAAGCACCTAAACATGATTTTTGGACATATTGGAGTGTATTGGGTGCGTTCGTGGCAAAAACTCACT
peak_40::Chr6:15427961-15428161	AGCACCTAAACATGGTTTTTGGACATATTGGAGTGTATTGGGTGCGTTCGTGGCAAAAACTCACTTCGCGACTCGCGCGGCGAACTTTTGTCAATTAATGCCGATATTGCCACTCGTGGGTGCGTTGTTTTTAACCGGAACGAAAAAGTTCCAAAAGCACCAAAACATGATTTTTGGACATATTGGATTGCATTGGGTGC
peak_41::Chr6:15433006-15433206	CCGATATTGCCACTCGTGGGTGCGTTGTTTTTAACCGGAACGAAAAAGTTCAAAAAGCACCAAAACATGGTTTTTGGACATATTGGAGTGTATTGGGTGCGTTCGTGGCAAAAACTCACTTCGCGACTCGCGCGGCGAACTTTTGCCAATTAATGCCGATATTGCCACTCGTGGGTGCGATGTAATATACCGGAATCAAA
peak_42::Chr6:15439962-15440162	AATGCCGATATTGCCACTCGTGGGTGCGTTGTTTTTAACCGAACGAAAAAGTGCAAAAAGCACCTAAACATGATTTTTGGACATATTGTACTGTATTGGGTGCGTTCGTGGCAAAAACTCACTTCGCGACTCGCGCGGCGAACTTTTGTCAATTAATGCCGATATTGCCACTCGTGGGTGCGTTGTTTTTAACTGGAACG
peak_43::Chr7:9904964-9905164	ACCCTGAGCTGTCCGTCCGTCTAGTCTCTGGGGACTATCCGGATTTTCAACGATTGGTGGACAAGAGCATCCGCTTGGAAGCCAAGCACAAGGAGCTGGAGTCGCACAAGCGCCGCTTGGCGAATTTCCGCAATCAACAGGGTGCTAACCAAAGGGTCCGCT

	ACACCAATCCCTATCCAGGGGGATCCTCCTCGCAGCAG
peak_44::Chr7:12163438-12163638	TTCAGTTCGGTTGAAAATATCGTACCCTTGTGTGCCAATAATGGCATTAATTGACAAAAGTTCGCCGCGCGAATCACGAAGTGAATTTTTGCCACGAACGCACCCTATACACTCCAATATGTCCAAAAAACATATTTGGTGCTTTTTGAACTTTTTCACTTCGGTCGAAAACATCGCACCTGTGTGCGCCAATATATGC
peak_45::Chr7:29697117-29697317	AGGGTTTAGGG
peak_46::Chr8:4937624-4937824	GGAACCCCCGGGACAGGCAGGCAGGACTGAGCCCCTAGCAGCAGGGCACCAACCCTCTGCCGTATGACATCTCGACTACCGGGCCGCAGCTCGTGTAGCCTTCATTTGCCCTGGAGAATGTCCATCGACCCCCGACTTCATCCATCTCCAATCCGTGTACTTTTGTTTATGACTAGCCTGAGCCACAAACTAAGCCTTAC
peak_47::Chr9:1359-1559	CAACGGGCGGCGGGCTGAATCCTTTGCAGACGACTTAAATACGCGACGGGGCATTGTAAGTGGCAGAGTGGCCTTGCTGCCACGATCCACTGAGATCCAGCCCCGCGTCGCACGGATTCGTCCCTCCCCCCTCTCCCCCGCGCCCCGCGCAGGTTCCCCCCGAGGCCGCCCCGGTCCGGCCAAGTCCCCAGGCCTCTCT
peak_48::Chr9:2945-3145	CTCCTAGCCTGCTGGCACGGCGCCCTGGCGAGTCTCGCCACGGGCCCGTTCCGCACGGTTTTTGAGGCACCCGTGCCGCCGAAAGAACGGGACTCGCTCCCGACACCTCTCCCACGCGTGGTGGCCCTCCGGTAGGCCGTCCTCCCAGCAGACCAGCCGTGCTCCGCGCGGCAGGATGCTTGGGCGGCCTTGCCGCCGTG
peak_49::Chr9:3747-3947	TGATGGTACGTGCTACTCGGATAACCGTAGTAATTCTAGAGCTAATACGTGCAACAAACCCCGACTTCCGGGAGGGGCGCATTTATTAGATAAAAGGCTGACGCGGGCTCCGCCCGCTGATCCGATGATTCATGATAACTCGACGGATCGCACGGCCCTCGTGCCGGCGACGCATCATTCAAATTTCTGCCCTATCAACT
peak_50::Chr9:4172-4372	CATTGGAGGGCAAGTCTGGTGCCAGCAGCCGCGGTAATTCCAGCTCCAATAGCGTATATTTAAGTTGTTGCAGTTAAAAAGCTCGTAGTTGGACCTTGGGCCGGGCCGGCCGGTCCGCCTCACGGCGAGCACCGACCTGCTCGACCCTTCTGCCGGCGATGCGCTCCTGGCCTTAACTGGCCGGGTCGTGCCTCCGGCGC
peak_51::Chr9:5033-5233	GGCCACGGAAGTTTGAGGCAATAACAGGTCTGTGATGCCCTTAGATGTTCTGGGCCGCACGCGCGCTACACTGATGTATCCAACGAGTATATAGCCTTGGCCGACAGGCCCGGGTAATCTTGGGAAATTTCATCGTGATGGGGATAGATCATTGCAATTGTTGGTCTTCAACGAGGAATGCCTAGTAAGCGCGAGTCATC
peak_52::Chr9:6156-6356	CGCCGTCCGAATTGTAGTCTGGAGAGGCGTCCTCAGCGACGGACCGGGCCCAAGTCCCCTGGAAAGGGGCGCCTGGGAGGGTGAGAGCCCCGTCCGGCCCGGACCCTGTCGCCCCACGAGGCGCCGTCAACGAGTCGGGTTGTTTGGGAATGCAGCCCAAATCGGGCGGTAAACTCCGTCCAAGGCTAAATACAGGCGAG
peak_53::Chr9:7575-7775	CGGCAGAGAGCGCGACCACGCGCGTGCCCCGAAAGGGAATCGGGTTAAGATTTCCCGAGCCGGGACGTGGCGGTTGACGGCGACGTTAGGAAGTCCGGAGACGCCGGCGGGGGCCTCGGGAAGAGTTATCTTTTCTGCTTAACGGCCCGCCAACCCTGGAAACGGTTCAGCCGGAGGTAGGGTCCAGCGGCCGGAAGAGC
peak_54::Chr9:9287-9487	CAACGGGCGGCGGGCTGAATCCTTTGCAGACGACTTAAATACGCGACGGGGCATTGTAAGTGGCAGAGTGGCCTTGCTGCCACGATCCACTGAGATCCAGCCCCGCGTCGCACGGATTCGTCCCTCCCCCCTCTCCCCCGCGCCCCGCGCAGGTTCCCCCC

	GAGGCCGCCCCGGTCCGGCCAAGTCCCCAGGCCTCTCT
peak_55::Chr9:10002-10202	CGAAAACTGTGTGCGAGCTGTGAAGGGCTGGACGCTAGGGGTGCGTTGGGCTGGCTATGGCCCTAGACTATAGTAGGGGGGAAGGGATGGCCGGGCTGCCACGCGCACGGCACCCGGTTCGGTCCACGTTCGGGCGCCGGGCCGACCGACCGGCACCCGTGCGCGAGTTGGGAAGGGCTGGCTCGTGCAGCCACCCACCG
peak_56::Chr9:10882-11082	TGCTGGCACGGCGCCCTGGCGAGTCTCGCCACGGGCCCGTTCCGCACGGTTTTTGAGGCACCCGTGCCGCCGAAAGAACGGGACTCGCTCCCGACACCTCTCCCACGCGTGGTGGCCCTCCGGTAGGCCGTCCTCCCAGCAGACCAGCCGTGCTCCGCGCGGCAGGATGCTTGGGCGGCCTTGCCGCCGTGGCTGCGTAG
peak_57::Chr9:12957-13157	TTTAGGCCACGGAAGTTTGAGGCAATAACAGGTCTGTGATGCCCTTAGATGTTCTGGGCCGCACGCGCGCTACACTGATGTATCCAACGAGTATATAGCCTTGGCCGACAGGCCCGGGTAATCTTGGGAAATTTCATCGTGATGGGGATAGATCATTGCAATTGTTGGTCTTCAACGAGGAATGCCTAGTAAGCGCGAGT
peak_58::Chr9:14087-14287	CGTCCGAATTGTAGTCTGGAGAGGCGTCCTCAGCGACGGACCGGGCCCAAGTCCCCTGGAAAGGGGCGCCTGGGAGGGTGAGAGCCCCGTCCGGCCCGGACCCTGTCGCCCCACGAGGCGCCGTCAACGAGTCGGGTTGTTTGGGAATGCAGCCCAAATCGGGCGGTAAACTCCGTCCAAGGCTAAATACAGGCGAGAGA
peak_59::Chr9:15265-15465	CGCTGAAGCGCGCGACCCACACCAGGCCATCTGGGCGAGCGCCATGCCCCGATGAGTAGGAGGGCGCGGCGGCCGCCGCAAAACCCGGGGCGCGAGCCCGGGCGGAGCGGCCGTCGGTGCAGATCTTGGTGGTAGTAGCAAATATTCAAATGAGAACTTTGAAGGCCGAAGAGGAGAAAGGTTCCATGTGAACGGCACTT
peak_60::Chr9:17196-17396	CGCCTCGAAGCTCCCTTCCCAACGGGCGGCGGGCTGAATCCTTTGCAGACGACTTAAATACGCGACGGGGCATTGTAAGTGGCAGAGTGGCCTTGCTGCCACGATCCACTGAGATCCAGCCCCGCGTCGCACGGATTCGTCCCTCCCCCCTCTCCCCCGCGCCCCGCGCAGGTTCCCCCCCGAGGCCGCCCCGGTCCGGC
peak_61::Chr9:18772-18972	GGTGTCGCACGTGAGATCCTTCCCACCGCCTCCTAGCCTGCTGGCACGGCGCCCTGGCGAGTCTCGCCACGGGCCCGTTCCGCACGGTTTTTGAGGCACCCGTGCCGCCGAAAGAACGGGACTCGCTCCCGACACCTCTCCCACGCGTGGTGGCCCTCCGGTAGGCCGTCCTCCCAGCAGACCAGCCGTGCTCCGCGCGG
peak_62::Chr9:22016-22216	GTCCGAATTGTAGTCTGGAGAGGCGTCCTCAGCGACGGACCGGGCCCAAGTCCCCTGGAAAGGGGCGCCTGGGAGGGTGAGAGCCCCGTCCGGCCCGGACCCTGTCGCCCCACGAGGCGCCGTCAACGAGTCGGGTTGTTTGGGAATGCAGCCCAAATCGGGCGGTAAACTCCGTCCAAGGCTAAATACAGGCGAGAGAC
peak_63::Chr9:23156-23356	CTCACCTGCCGAATCAACTAGCCCCGAAAATGGATGGCGCTGAAGCGCGCGACCCACACCAGGCCATCTGGGCGAGCGCCATGCCCCGATGAGTAGGAGGGCGCGGCGGCCGCCGCAAAACCCGGGGCGCGAGCCCGGGCGGAGCGGCCGTCGGTGCAGATCTTGGTGGTAGTAGCAAATATTCAAATGAGAACTTTGAA
peak_64::Chr9:25869-26069	TGCGAGCTGTGAAGGGCTGGACGCTAGGGGTGCGTTGGGCTGGCTATGGCCCTAGACTATAGTAGGGGGGAAGGGATGGCCGGGCTGCCACGCGCACGGCACCCGGTTCGGTCCACGTTCGGGCGCCGGGCCGACCGACCGGCACCCGTGCGCGAGTTGGGAAGGGCTGGCTCGTGCAGCCACCCACCGGCCGACCGACC
peak_65::Chr9:26730-26930	TCCTAGCCTGCTGGCACGGCGCCCTGGCGAGTCTCGCCACGGGCCCGTTCCGCACGGTTTTTGAGGCACCCGTGCCGCCGAAAGAACGGGACTCGCTCCCGACACCTCTCCCACGCGTGGTGGCCCTCCGGTAGGCCGTCCTCCCAGCAGACCAGCCGTGC

	TCCGCGCGGCAGGATGCTTGGGCGGCCTTGCCGCCGTGG
peak_66::Chr9:28810-29010	CCGTTTAGGCCACGGAAGTTTGAGGCAATAACAGGTCTGTGATGCCCTTAGATGTTCTGGGCCGCACGCGCGCTACACTGATGTATCCAACGAGTATATAGCCTTGGCCGACAGGCCCGGGTAATCTTGGGAAATTTCATCGTGATGGGGATAGATCATTGCAATTGTTGGTCTTCAACGAGGAATGCCTAGTAAGCGCG
peak_67::Chr9:29943-30143	CGTCCGAATTGTAGTCTGGAGAGGCGTCCTCAGCGACGGACCGGGCCCAAGTCCCCTGGAAAGGGGCGCCTGGGAGGGTGAGAGCCCCGTCCGGCCCGGACCCTGTCGCCCCACGAGGCGCCGTCAACGAGTCGGGTTGTTTGGGAATGCAGCCCAAATCGGGCGGTAAACTCCGTCCAAGGCTAAATACAGGCGAGAGA
peak_68::Chr9:31371-31571	CGACCACGCGCGTGCCCCGAAAGGGAATCGGGTTAAGATTTCCCGAGCCGGGACGTGGCGGTTGACGGCGACGTTAGGAAGTCCGGAGACGCCGGCGGGGGCCTCGGGAAGAGTTATCTTTTCTGCTTAACGGCCCGCCAACCCTGGAAACGGTTCAGCCGGAGGTAGGGTCCAGCGGCCGGAAGAGCACCGCACGTCGC
peak_69::Chr9:34694-34894	CCACGGGCCCGTTCCGCACGGTTTTTGAGGCACCCGTGCCGCCGAAAGAACGGGACTCGCTCCCGACACCTCTCCCACGCGTGGTGGCCCTCCGGTAGGCCGTCCTCCCAGCAGACCAGCCGTGCTCCGCGCGGCAGGATGCTTGGGCGGCCTTGCCGCCGTGGCTGCGTAGCGTATGAGCAGCTTTGGACCGGTGTATG
peak_70::Chr9:35879-36079	GGATCCATTGGAGGGCAAGTCTGGTGCCAGCAGCCGCGGTAATTCCAGCTCCAATAGCGTATATTTAAGTTGTTGCAGTTAAAAAGCTCGTAGTTGGACCTTGGGCCGGGCCGGCCGGTCCGCCTCACGGCGAGCACCGACCTGCTCGACCCTTCTGCCGGCGATGCGCTCCTGGCCTTAACTGGCCGGGTCGTGCCTCC
peak_71::Chr9:36741-36941	TTTAGGCCACGGAAGTTTGAGGCAATAACAGGTCTGTGATGCCCTTAGATGTTCTGGGCCGCACGCGCGCTACACTGATGTATCCAACGAGTATATAGCCTTGGCCGACAGGCCCGGGTAATCTTGGGAAATTTCATCGTGATGGGGATAGATCATTGCAATTGTTGGTCTTCAACGAGGAATGCCTAGTAAGCGCGAGT
peak_72::Chr9:2775840-2776040	TTGTATTGGGTGCGTTCGCGGCAAAAACTCAGTTCGTGATTCGCGTGGCGAACTTTTGTCAATTAATGCTATTATTGGCCCACGTTGGTACGATATTTTCGACCGAAATGAAAAAGTTCAAATAACTCCAAACCATGATTTTTTGCACATATTGGAGTGTATTGGGTGCGTTCGTGGCAAAAACTCCCTACGTGATTCGC
peak_73::Chr9:2851129-2851329	TTTTTGTAAATAATGGATTGTATTGGGTGCGTTCGCGGCAAAAACTCAGTTCGTGATTCGCGTGGCGAACTTTTGTCCATTAATGCTATTATTGGCCCACGTGGGTACGATATTTTCGACCGAAATGAAAAAGTTCAAATAACTCCAAACCATGATTTTTGCACATATTGGAGTGTATTGGGTGCGTTCGTGGCAAAAAC
peak_74::ChrSy:370710-370910	CACCCAATACACTCCAATATGTCCAAAAATCATGTTTTGGTGCATTTTTGAACTTTTTCATTCCGGTCAAAAACATCGCACCCGTGTGGGCCAATATTGGCATTAATTGACAAAAGTTCGCCGCGTGTATCACGAAGTGAGTTTTTGCCACGAACGCACCCAATACACTCCAATATGTCCGAAAATCATGTTTTGGAGCT
peak_75::ChrSy:371654-371854	TACCGAATACACTCCAATATGTCCAAAAATCATGCTTTGGTGCTTTTTTGAACTTTTTCATTACGGACAAAAACATCGCACCCGTGTGGGCCAATATTGGCATTAATTGACAAAAGTTCGCCGCGTGTATCACGAAGTGAGTTTTTGCCACGAACGCACCCAATACACTCCAATATGTCCAAAAATCATGTTTTGGTGAT
