# Supplementary material for: MYB57 transcriptionally regulates MAPK11 to interact with PAL2;3 and modulate rice allelopathy
Source: J Exp Bot. 2019 Dec 7;71(6):2127–41. doi: 10.1093/jxb/erz540 (PMC7242072; doi:10.1093/jxb/erz540)
Supplement: erz540_suppl_Supplementary_Figure_S1 [file erz540_suppl_supplementary_figure_s1.pdf]

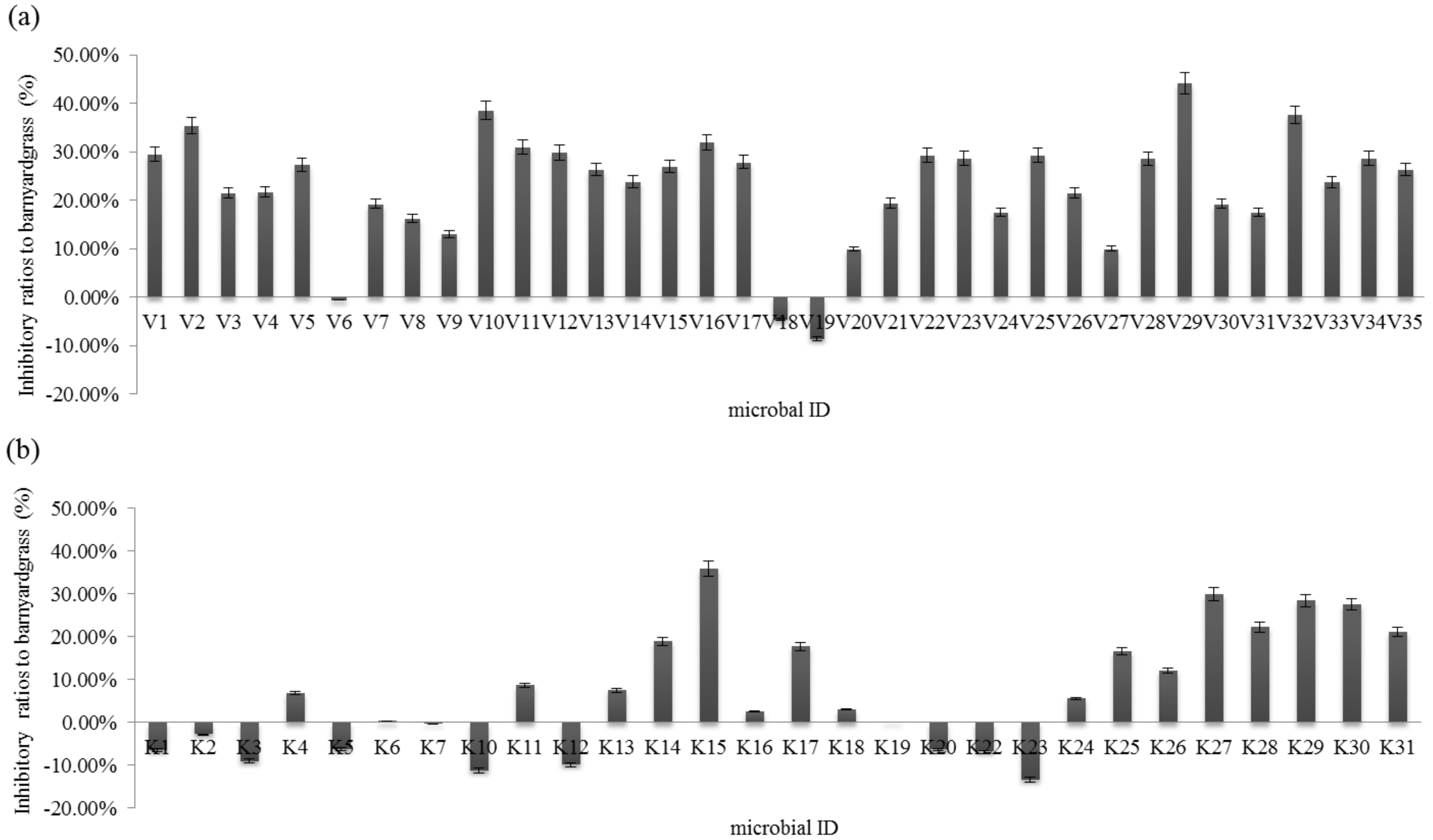

Fig. S1. The allelopathic inhibitory ratios (IRs) to barnyardgrass from specific microbial strains isolated from the rhizospheric soil of Kitaake and *Os*MYB57$_{VP64}$. Thirty-one and 35 microbial strains were isolated from the rhizospheric soil of *Os*MYB57$_{vp64}$ and Kitaake respectively, and their IRs to the barnyardgrass were detected using their fermentation broth. K1-K31, 31 individual strains isolated from the rhizospheric soil of Kitaake and their IRs to the barnyardgrass (a); V1-V35, 35 individual strains isolated from the rhizospheric soil of *Os*MYB57$_{vp64}$ and their IRs to the barnyardgrass (b).
