# Supplementary material for: MYB57 transcriptionally regulates MAPK11 to interact with PAL2;3 and modulate rice allelopathy
Source: J Exp Bot. 2019 Dec 7;71(6):2127–41. doi: 10.1093/jxb/erz540 (PMC7242072; doi:10.1093/jxb/erz540)
Supplement: erz540_suppl_Supplementary_Table_S1-S4 [file erz540_suppl_supplementary_table_s1-s4.pdf]

## Table S1. Primers used in this study

| | Gene | Forward primer (5'-3') | Reverse primer (5'-3') |
|---|---|---|---|
| ChIP-qPCR | *OsRTP* | ACGGGTGCGATGTTTTTGAC | CCACGAACGCACCCAATACA |
| | *OsMAPK11* | TATTGGAGTGTATTGGGTGGGTT | TAGGTGCTTTTTGAACTTTTTGAT |
| | *OsPAL2;3* | TGCGTTACCTCAGTATGCC | GAGAGGGAAAGGGAAAAGG |
| | *OsKSL4* | CGCAGATTCCGTCTTCAA | CAGGGATGCAATGCACTTA |
| *Promoter region of OsPAL2;3* (For activity detection) | *OsPAL2;3* | *atgattacgaattcgagctc*GGTACTGGTACCGCCAGTACGCATA | *tgtagtccatactagt*AGTTAGCTAGACGGCCGGAGAGAAC |
| *Promoter region of OsPAL2;3* (For DNA – pull down) | *OsPAL2;3* | (5' biotin labeled )GGTACTGGTACCGCCAGTACGCATA | AGTTAGCTAGACGGCCGGAGAGAAC |
| qPCR | *OsPAL* | CCGTGCTCTTTGAGGCTAAC | GCTTGTGAGTCAGGTGGTCG |
| | *OsC4H* | ACCGCAGCGTCTCCTTC | ACCACCCGAGCATCCAG |
| | *OsCOL* | TGGTGGAGTGCGTGCTG | AGGCGTTGGCGTAGATG |
| | *OsOMT* | TGTCCTGTGAAATGGGTG | CCTCGGAACAAGAACTG |
| | *OsCCA* | TGGGAGCAGGAATGGCAAAAT | TTCCGTCCCACCAGCATGAC |
| | *OsCAD* | TCGGCGTCGCTAATTTCATCC | TCGATGGAAGAACGGGCAGAG |
| | *OsCPS4* | CCATCGAGATTTTTGGAGGA | TGGAAAGTTTGCAGCAGATG |
| | *OsKSL4* | ATGGGTTGCTGGTCAGGTAG | GTTCCAGCGTGGCATAAAAT |
| | *OsMAS* | GAAGCTCGACGTCATGTTCA | GCTCGAAGTCCTCCTTGGT |
| | *OsWHY1* | AGGAGGGTTTCTTCCGTCTG | TCCGCACTTTACCTTCATCAC |
| | *OsWHY2* | TGTCACCTACTGAAGTCGGAAGC | ATGCGAAACTCAATGCTGTGC |
| | *Oshistone H4* | ACATCCAGGGGATCACGAAG | GCCGAAGCCGTAGAGGGT |
| | *β-actin* | CTGCGGGTATCCATGAGACT | GCAATGCCAGGGAACATAGT |
| BiFC | *OsMAPK11* | *atggcgcgccactagt*ATGCAGACCAGCAATTTTCGTAAG | *cacctcctccactagt* GGTGATTCTATACGTCCCTGGAAGA |
| | *OsPAL2;3* | *atggcgcgccactagt*ATGGCGTGCGAGAACGGTCAG | *cacctcctccactagt*GCAGATGGGCAGGGGCGCGC |
| Co-IP | *OsMAPK11* | *gacaagacgcgtcccggg*ATGCAGACCAGCAATTTTCGTAAG | *gagaaagcttggatcc*GGTGATTCTATACGTCCCTGGAAGA |
| | *OsPAL2;3* | *tgacctcgagactagt*ATGGCGTGCGAGAACGGTCAG | *aggtggaggtcccccggg*GCAGATGGGCAGGGGCGCGC |

**Table S2. The relative contents of secondary metabolites detected from the root tissue of *Os*MYB57$_{vp64}$ and Kitaake**

| Compound | molecular formula | Kitaake | *Os*MYB57$_{vp64}$ |
|---|---|---|---|
| | | Peak area | Peak area |
| Benzeneacetic acid | $C_8H_8O_2$ | 114365345a | 142990703a |
| *p*-Coumaric acid | $C_9H_8O_3$ | 107528557a | 61744259b |
| L-phenylalanine | $C_9H_{11}NO_2$ | - | 42605272a |
| *p*-toluic acid | $C_8H_8O_2$ | - | 21614622a |
| Dimethylmalonic acid | $C_5H_8O_4$ | 4431598a | 43674383a |

Note: the data shows mean value of three independent determination.

**Table S3. High allelopathic microbes isolated from the rhizospheric soil of *Os*MYB57$_{vp64}$ and Kitaake**

| ID of strains | Microbe alignment from Genbank |
|---|---|
| V2 | *Pseudomonas* spp. YXE3-18 |
| V10 | *Bacillus* spp. B-15 |
| V11 | *Pseudomonas* spp. J3.2C5 |
| V16 | *Penicillium aculeatum* strain H23 |
| V29 | *Penicillium rubidurum* isolate CY249 |
| V32 | *Streptomyces viridobrunneus* strain SCPE-09 |
| K15 | *Streptomyces* spp. FXJ1.430 |

**Table S4. Motifs from the sequence of peaks**

| Motif ID | Motif logo | E-value | Sites | Width |
|---|---|---|---|---|
| 1 | 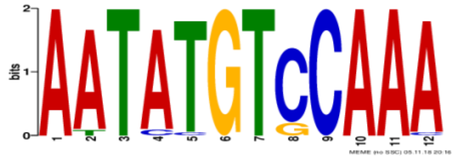 | 9.9e-096 | 33 | 12 |
| 2 | 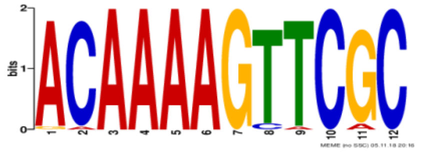 | 1.8e-087 | 31 | 12 |
| 3 | 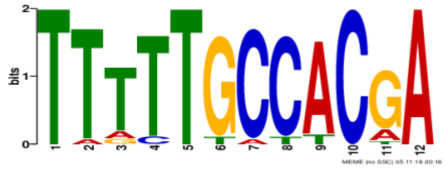 | 2.4e-090 | 39 | 12 |
| 4 | 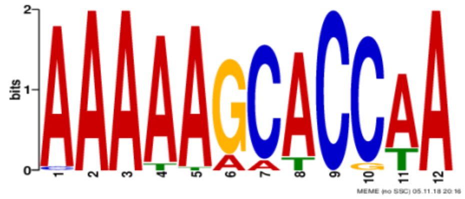 | 3.0e-081 | 34 | 12 |
| 5 | 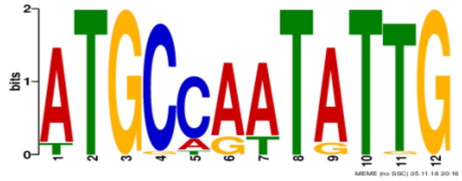 | 3.9e-075 | 34 | 12 |
| 6 | 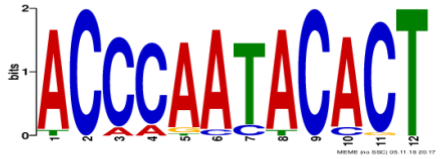 | 2.4e-073 | 33 | 12 |
| 7 | 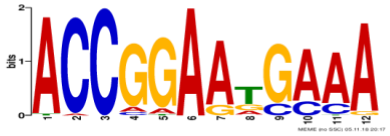 | 4.0e-068 | 41 | 12 |
| 8 | 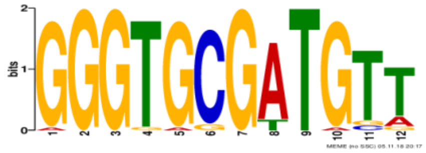 | 1.5e-064 | 31 | 12 |

| | | | | |
|---|---|---|---|---|
| 9 | 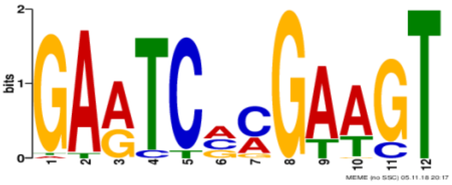 | 1.4e-051 | 42 | 12 |
| 10 | 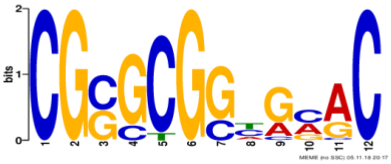 | 1.3e-024 | 39 | 12 |
| 11 | 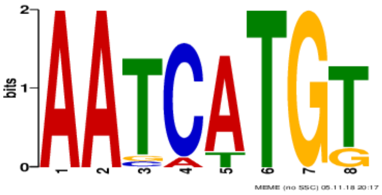 | 3.8e-021 | 36 | 8 |
| 12 | 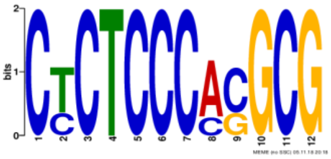 | 3.5e-009 | 12 | 12 |
| 13 | 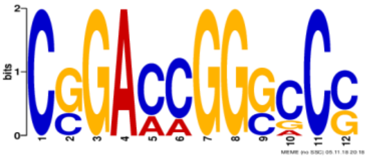 | 7.0e-009 | 18 | 12 |
| 14 | 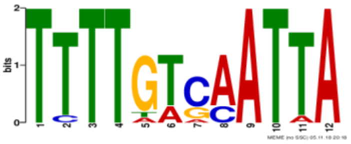 | 1.3e-008 | 12 | 12 |
| 15 | 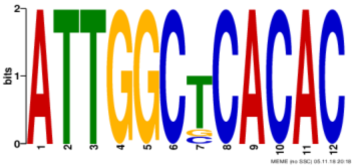 | 3.3e-008 | 9 | 12 |

Note: Sites indicate the number of sites contributing to the construction of the motif.
