# Supplementary material for: MYB57 transcriptionally regulates MAPK11 to interact with PAL2;3 and modulate rice allelopathy
Source: J Exp Bot. 2019 Dec 7;71(6):2127–41. doi: 10.1093/jxb/erz540 (PMC7242072; doi:10.1093/jxb/erz540)
Supplement: erz540_suppl_Supplementary_Legends [file erz540_suppl_supplementary_legends.docx]

**Supplementary data**

Table S1. Primers used in this study.

Table S2. The relative contents of secondary metabolites detected from the root tissue of *Os*MYB57vp64 and Kitaake.

Table S3. High allelopathic microbes isolated from the rhizospheric soil of *Os*MYB57_vp64_ and Kitaake.

Table S4. Motifs from the sequence of peaks.

Fig. S1. The allelopathic inhibitory ratios (IRs) to barnyardgrass from specific microbial strains isolated from the rhizospheric soil of Kitaake and *Os*MYB57_VP64_.

Dataset S1. Sequence of 16srDNA or ITS from the specific microbial strains with allelopathic inhibitory ratios to barnyardgrass.

Dataset S2. Sequence of peaks from ChIP-seq.
